# Spectrum of disease-causing mutations in protein secondary structures

**DOI:** 10.1186/1472-6807-7-56

**Published:** 2007-08-29

**Authors:** Sofia Khan, Mauno Vihinen

**Affiliations:** 1 Institute of Medical Technology, FI-33014 University of Tampere, Finland; 2 Research Unit, Tampere University Hospital, FI-33520 Tampere, Finland

## Abstract

**Background:**

Most genetic disorders are linked to missense mutations as even minor changes in the size or properties of an amino acid can alter or prevent the function of the protein. Further, the effect of a mutation is also dependent on the sequence and structure context of the alteration.

**Results:**

We investigated the spectrum of disease-causing missense mutations in secondary structure elements in proteins with numerous known mutations and for which an experimentally defined three-dimensional structure is available. We obtained a comprehensive map of the differences in mutation frequencies, location and contact energies, and the changes in residue volume and charge – both in the mutated (original) amino acids and in the mutant amino acids in the different secondary structure types. We collected information for 44 different proteins involved in a large number of diseases. The studied proteins contained a total of 2413 mutations of which 1935 (80%) appeared in secondary structures. Differences in mutation patterns between secondary structures and whole proteins were generally not statistically significant whereas within the secondary structural elements numerous highly significant features were observed.

**Conclusion:**

Numerous trends in mutated and mutant amino acids are apparent. Among the original residues, arginine clearly has the highest relative mutability. The overall relative mutability among mutant residues is highest for cysteine and tryptophan. The mutability values are higher for mutated residues than for mutant residues. Arginine and glycine are among the most mutated residues in all secondary structures whereas the other amino acids have large variations in mutability between structure types. Statistical analysis was used to reveal trends in different secondary structural elements, residue types as well as for the charge and volume changes.

## Background

Now that the sequence of the human genome is almost complete, the research interest in genomics has moved from determining the sequence to the analysis of genetic variations, e.g. the Human Variome Project [1], and collecting data in locus-specific mutation databases [2,3]. There is also a race to develop methods for cost-effective sequencing of the genomes of individuals [4]. Missense mutations in coding DNA, which lead to single amino acid changes in proteins, are commonly linked to human disorders [5]. The number of documented disease-linked missense and nonsense mutations is close to 30,000 [6]. A disease phenotype can arise because an amino acid change results in the loss of a critical protein function, in structural alterations, or because the mutation leads to "gain of function" effects such as functional dysregulation or the formation of toxic aggregates [7-9].

Only correctly folded proteins can deliver all the functional properties of a protein. Even minor changes in the size or properties of an amino acid side chain can alter or prevent the function of the protein. On the other hand, even large deletions or insertions may be tolerated in numerous positions within a protein [10]. The effects of mutations are also dependent on the protein sequence and structure context of the alteration. General statistical analyses have been performed for disease-causing mutations, for non-synonymous SNPs (nsSNPs) [9,11-16], for groups of diseases, such as immunodeficiencies [3], and for groups of proteins, such as protein kinases [17]. Based on these studies and others, a number of methods have been developed for the prediction of tolerance and the consequences of mutations [13,18-23].

Structural information is needed to fully understand the effects and consequences of mutations, whether disease-causing or used purposefully to modify the properties of a protein e.g. in protein engineering. Three-dimensional structures and computer models have been used by us and others to elucidate disease mechanisms from amino acid substitutions e.g. in references [24-26]. We have reviewed and discussed the applicability of more than 30 sequence and structure utilizing methods to predict the outcome of missense mutations to explain the basis of diseases [27]. Activity modifying mutations are also valuable for understanding the functions and conservation of amino acids and sequence regions in protein families. Recently, all the possible amino acid substitutions and their effects on biophysical properties were investigated in five proteins [28].

Secondary structural elements, α-helices, β-strands, turns and bends, are basic structural components of protein scaffolds. Amino acids are differently distributed between these elements. This information has been utilized for decades to predict the location of secondary structures from sequence information e.g. in references [29-34]. Secondary structures are common regular conformations of polypeptides, and they are the most energetically favourable structures. Each secondary structure type has characteristic backbone φ and ψ angles. Secondary structures fold together in proteins and form higher order structures such as super secondary structures, motifs, domains and tertiary structures. The organization of the folds is very similar in related protein structures even though the sequence identities can be very small. Secondary structures are generally of substantial length and can pass through the hydrophobic core of globular proteins. Within protein families the secondary structures are more conserved than the surface loops connecting the adjacent elements.

Since secondary structures are structural building blocks that cover some 25 – 75% of the length of proteins, it would be interesting to know how disease-related, and thus function and/or structure altering, mutations affect these elements. We investigated the occurrence, location and distribution of disease-causing mutations in secondary structures. The study is based on statistics and bioinformatical analysis of three-dimensional structures and protein sequence information. Not many differences occur in mutation types between secondary structure elements and whole proteins. Clear differences were observed within the mutation spectra for different secondary structural elements and regions outside secondary structures. Some features, like the overrepresentation of arginines, were evident in all the secondary structures. Our analysis covers different amino acid substitutions, alterations of physicochemical properties, and sequence conservation. We investigated mutations both in the mutated original residues and in the mutant, altered, amino acids.

## Results and discussion

**Figure 1:**
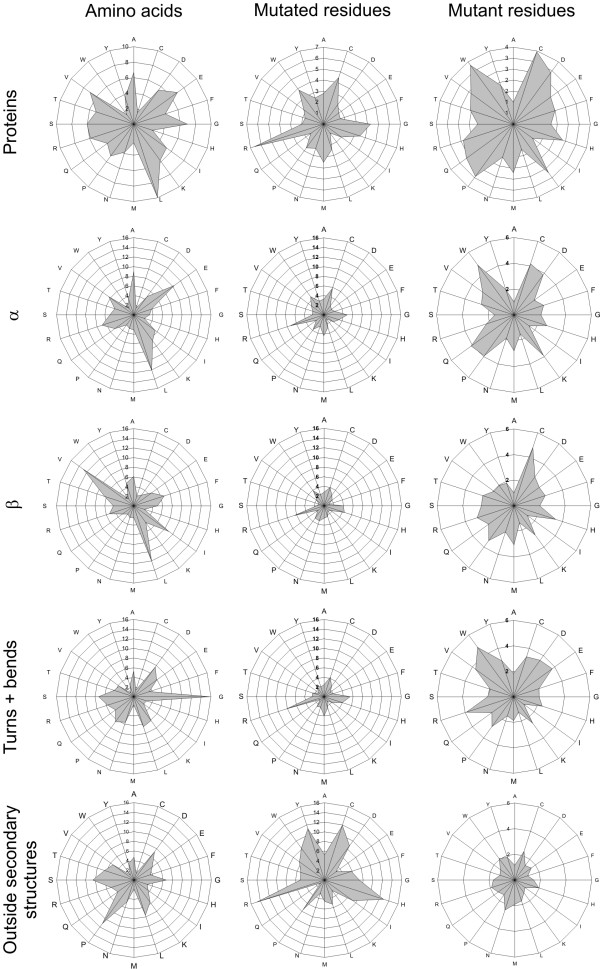
**Amino acid distribution and relative mutability**. Top row. Amino acid distribution in the investigated proteins (left), overall relative mutability of mutated (middle) and mutant residues (right). The same information is on the second row only for α-helices, third row for β-strands, fourth row for turns and bends, and in the bottom row for structures outside secondary structural elements.

Secondary structures have an energetically favourable organization of the polypeptide chain. Our aim was to obtain a comprehensive map of the differences in mutation frequencies, location, contact energies and changes in residue volume and charge, both in the mutated amino acids and in the mutant amino acids, for the different secondary structure types. We collected information for 44 proteins involved in a large number of diseases (Table 1). The criteria for choosing the proteins were a relatively large number of reported missense mutations and the availability of the three-dimensional structure. The genes in Table 1 are listed with the recommended HGNC names (HUGO Gene Nomenclature Committee) [35,36]. The number of missense mutations varied from 8 to 240 per investigated protein or domain. The proteins represent different activities and functions including enzymes, signalling proteins, membrane proteins, receptors etc. 42 of the total of 46 structures had a resolution greater than 2.00 Å. There are more PDB entries than proteins because for the large BTK and VDR proteins there are two structures for different domains. The studied proteins contained altogether 2413 mutations of which 1935 (80%) appeared in secondary structures. The amino acid composition of all the proteins is in Figure 1. Considering the large size of the dataset and diversity of protein types and functions the statistically significant results reveal the true nature of disease-causing amino acid changes. In the χ^2^-test, results were considered significant with a *P *value < 0.05.

**Table 1:** Summary of analysed proteins and diseases

Name	Symbol	PDB	OMIM	Disease(s)	Mutations
ABO blood group (transferase A, α1–3-N-acetylgalactosaminyltransferase; transferase B, α1–3-galactosyltransferase)	ABO	1lzj	110300	Blood group variation	22
alanine-glyoxylate aminotransferase (oxalosis I; hyperoxaluria I; glycolicaciduria; serine-pyruvate aminotransferase)	AGXT	1h0c	604285	Hyperoxaluria	22
arylsulfatase B	ARSB	1fsu	253200	Mucopolysaccharidosis VI	30
argininosuccinate lyase	ASL	1k62	608310	Argininosuccinate lyase deficiency	21
Bruton agammaglobulinemia tyrosine kinase	BTK	1btk, 1k2p	300300	X-linked agammaglobulinaemia	144
cystathionine-β-synthase	CBS	1jbq	236200	Homocystinuria	74
CD40 ligand (TNF superfamily, member 5, hyper-IgM syndrome)	CD40LG	1aly	300386	Hyper-IgM syndrome	27
cystic fibrosis transmembrane conductance regulator, ATP-binding cassette (sub-family C, member 7)	CFTR	1xmj	602421	Asthma; Cystic fibrosis; Idiopathic pancreatitis; Congenital absence of vas deferens; Hereditary pancreatitis; Hypertrypsinaemia; Primary sclerosing cholangitis; Susceptibility to sarcoidosis; Idiopathic Bronchiectasis	86
CHK2 checkpoint homolog	CHEK2	1gxc	604373	Multiple cancers	10
doublecortex; lissencephaly, X-linked (doublecortin)	DCX	1mjd	300121	Double cortex syndrome; X linked lissencephaly syndrome; Subcortical band heterotopia; Resistant partial seizures	16
cytochrome b5 reductase 3	CYB5R3	1umk	250800	Methaemoglobinaemia	18
ectodysplasin A	EDA	1rj7	300451	Ectodermal dysplasia	22
coagulation factor XIII, A1 polypeptide	F13A1	1f13	134570	Factor XIII deficiency	30
coagulation factor VIII, procoagulant component (hemophilia A)	F8	1iqd	306700	Haemophilia A	39
glucose-6-phosphate dehydrogenase	G6PD	1qki	305900	Glucose-6-phosphate dehydrogenase deficiency	130
GTP cyclohydrolase 1 (dopa-responsive dystonia)	GCH1	1fb1	600225	Dopa-responsive and progressive dystonia; Tetrahydrobiopterin deficiency	45
galactosidase, α	GLA	1r46	301500	Fabry disease	179
glucuronidase, β	GUSB	1bhg	253220	Mucopolysaccharidosis VII; Hydrops fetalis	28
hemoglobin, β	HBB	1o1p	141900	Haemoglobin variant; Haemolytic anaemia; β-thalassaemia; Erythrocytosis; Sickle cell anaemia	160
homogentisate 1,2-dioxygenase (homogentisate oxidase)	HGD	1eyb	607474	Alkaptonuria	29
hypoxanthine phosphoribosyltransferase 1 (Lesch-Nyhan syndrome)	HPRT1	1bzy	308000	Hypoxanthine guanine phosphoribosyltransferase deficiency; Lesch-Nyhan syndrome; Hyperuricaemia; Hyperuricaemia with neurologic symptoms	105
insulin receptor	INSR	1ir3	147670	Leprechaunism; Insulin resistance; Insulin resistance A; Association with reduced diastolic blood pressure; Diabetes, NIDDM; Rabson-Mendenhall syndrome	19
lamin A/C	LMNA	1ifr	150330	Muscular dystrophy; Emery-Dreifuss; Seip syndrome; Dilated cardiomyopathy; Partial lipodystrophy; Atypical Werner Syndrome; Familial autosomal dominant partial lipodystrophy (Dunnigan variety); Hutchinson-Gilford progeria syndrome; Charcot-Marie-Tooth disease 2; Cardiac conduction defects; Muscular dystrophy; Partial lipodystrophy; Mandibuloacral dysplasia; Association with metabolic syndrome	22
neurofibromin 2 (bilateral acoustic neuroma)	NF2	1h4r	607379	Neurofibromatosis 2	8
ornithine aminotransferase (gyrate atrophy)	OAT	2oat	258870	Gyrate atrophy	27
ornithine carbamoyltransferase	OTC	1oth	300461	Ornithine transcarbamylase deficiency; Hyperammonaemia	154
phenylalanine hydroxylase	PAH	1j8u	261600	Phenylketonuria; Hyperphenylalaninaemia	240
paired box gene 6 (aniridia, keratitis)	PAX6	6pax	607108	Aniridia; Congenital cataract; Peters' anomaly; Optic-nerve malformations; Congenital nystagmus; Ectopia pupillae; Isolated foveal hypoplasia; Ocular anterior segment anomaly	27
pyruvate dehydrogenase (lipoamide) α1	PDHA1	1ni4	300502	Pyruvate dehydrogenase deficiency; Lactic acidosis; Leigh syndrome	40
pyruvate kinase, liver and RBC	PKLR	1liu	266200	Elevated red cell ATP; Haemolytic anaemia; Pyruvate kinase deficiency	93
prion protein (p27–30) (Creutzfeldt-Jakob disease, Gerstmann-Strausler-Scheinker syndrome, fatal familial insomnia)	PRNP	1i4m	176640	Gerstmann-Straussler-Scheinker syndrome; Dementia; Creutzfeld-Jakob syndrome; Schizophrenia; Familial spongiform encephalopathy; Fatal familial insomnia; Prion disease	23
retinoblastoma 1 (including osteosarcoma)	RB1	1gux	180200	Retinoblastoma	18
solute carrier family 4, anion exchanger, member 1 (erythrocyte membrane protein band 3, Diego blood group)	SLC4A1	1hyn	109270	Spherocytosis; Blood group variation; Erythrocyte band 3 deficiency; Anaemia; Distal renal tubular acidosis; Acanthocytosis	7
superoxide dismutase 1, soluble (amyotrophic lateral sclerosis 1 (adult))	SOD1	1mfm	147450	Amyotrophic lateral sclerosis; Motor neuron disease	69
sex determining region Y	SRY	1j46	480000	XY sex reversal; Gonadal dysgenesis; Hermaphroditism	31
transcription factor 1, hepatic; LF-B1, hepatic nuclear factor (HNF1), albumin proximal factor	TCF1	1ic8	142410	Diabetes mellitus, type 2; MODY; MODY2; MODY3; Serum C-peptide and insulin response; Insulin resistance	40
thyroid hormone receptor, β thyroid hormone receptor, beta (erythroblastic leukemia viral (v-erb-a) oncogene homolog 2, avian)	THRB	1nq2	190160	Thyroid hormone resistance	72
troponin T type 2 (cardiac)	TNNT2	1j1d	191045	hypertrophic/dilated cardiomyopathy	4
troponin I type 3 (cardiac)	TNNI3	1j1e	191044	hypertrophic/restrictive cardiomyopathy	7
tumor protein p53 (Li-Fraumeni syndrome)	TP53	1tup	191170	Li-Fraumeni syndrome; Adrenocortical carcinoma; Sarcoma; Lung cancer; Breast cancer; Carcinoma; Glioma; Astrocytoma; Adrenocortical carcinoma; Glioblastoma; Cytosarcoma phyllodes; Osteosarcoma; Multiple cancers; Familial adenomatous polyposis; Rhabdomyosarcoma; Ependymoma; Adenocarcinoma; Thyroid tumour; Leukaemia/lymphoma; Neuroblastoma	73
uroporphyrinogen decarboxylase	UROD	1r3s	176100	Porphyria cutanea tarda; Hepatoerythropoietic porphyria	37
uroporphyrinogen III synthase (congenital erythropoietic porphyria)	UROS	1jr2	606938	Erythropoietic porphyria; Guenther disease	21
vitamin D (1,25-dihydroxyvitamin D3) receptor	VDR	1ie9, 1kb2	601769	Higher bone mineral density; Rickets	10
von Hippel-Lindau tumor suppressor	VHL	1lm8	608537	von Hippel-Lindau syndrome; Phaeochromocytoma; Haemangioblastoma; Pancreatic cancer; Polycythemia, with high erythropoietin concentration; Phaeochromocytoma and paraganglioma	134

The total chain length of the 44 investigated proteins is 12540 amino acids. The secondary structure elements consist as follows: all helix structures altogether 4567 amino acids of which α-helices 4118 (~90%), 3_10_-helices 449 (~10%) and π-helices just 2 residues. There are 153 amino acids in β-bridges and 2530 in β-ladders, altogether 2683 (27%), and there are 1436 (15%) and 1190 (12%) residues in turns and bends, respectively. 18 proteins belong to SCOP [37] class for all-α proteins, 12 proteins belong to all-β proteins, 22 proteins belong to α and β proteins (14 α/β and 8 α+β) and 2 to coiled coil proteins. Btk PH domain belongs to all-β proteins and kinase domain to α+β proteins. In VDR, nuclear receptor ligand-binding domain belongs to all-α proteins and glucocorticoid receptor-like (DNA-binding domain) to SCOP class for small proteins. Altogether there were 1939 mutations in the secondary structures, 48% in helices, 28% in β-structures, and 24% in turns and bends. These numbers follow the distribution of the structure elements. The length of the secondary structures varied as follows: α-helices from 1 to 46 residues, average length being 11.3 residues, 3_10_-helices from 2 to 9 residues, average 3.4. In β-strands the length varied from 1 to 22, average 5.28. Length of the structure in turns varied from 1 to 8 residues, average 2.1, and in bends from 1 to 6, average 1.6.

### Overall amino acid mutational spectrum

Table 2 summarizes all the mutations in the studied proteins and Table 3 mutations in the secondary structures only. DSSP classifies secondary structures into three helix types, two extended β-strand types, turns and bends. The helices represent the classical α-helix, π-helix and 3_10_-helix. The difference between the helix types originates from the number of residues per turn. In the normal right-handed α-helix there are 3.6 residues per turn, while π-helix has 4.4, and 3_10_-helix only three. In helical structures the main chain residues form stabilizing hydrogen bonds with residues further in the sequence. All the side chains are pointing out from the helical core. A large proportion of α-helices are amphipathic i.e. one face of the helix is hydrophilic and the other hydrophobic [38]. The 3_10_-helix is the fourth most common type of secondary structure in proteins after α-helices, β-strands, and reverse turns [39]. 3_10_-helices commonly appear as N- or C-terminal extensions of α-helices. They are typically only three residues long compared with a mean of 10–12 residues for α-helices [40]. β-Strands are divided into isolated β-bridges and extended strands. β-Strands can form structure stabilizing hydrogen bonds only with another strand in a parallel or antiparallel manner. Bends and turns are, according to DSSP terminology, relatively short structures that are located between the helical and strand elements. Well organized β- and γ-turns reverse the direction of the polypeptide chain. Only glycines are allowed in certain turns at certain positions due to steric restrictions in very tight bends.

**Table 2:** Spectrum of mutations in all residues in the studied proteins^a^

Amino acid	Original residues	Expected residues	χ^2^	*P *value	Mutant residues	Expected residues	χ^2^	*P *value
A	169	161	0.43	5.14E-01	83	148	28.28***	1.05E-07
C	79	38	43.38***	4.51E-11	133	86	25.54***	4.34E-07
D	98	131	8.28**	4.00E-03	155	98	32.54***	1.16E-08
E	113	168	17.82***	2.43E-05	84	86	0.05	8.20E-01
F	66	104	13.66***	2.19E-04	90	98	0.72	3.97E-01
G	246	167	37.28***	1.02E-09	106	141	8.89**	2.87E-03
H	87	61	10.74***	1.05E-03	108	98	0.93	3.34E-01
I	99	126	5.76*	1.64E-02	80	129	18.71***	1.52E-05
K	56	140	50.05***	1.50E-12	109	86	6.09*	1.36E-02
L	231	239	0.29	5.89E-01	113	203	39.88***	2.70E-10
M	78	58	7.00**	8.15E-03	97	55	31.33***	2.18E-08
N	90	92	0.03	8.58E-01	74	98	6.05**	1.39E-02
P	94	122	6.54**	1.05E-02	194	148	14.58***	1.35E-04
Q	62	102	15.50***	8.24E-05	115	86	9.69**	1.85E-03
R	348	142	299.39***	4.48E-67	222	209	0.79	3.73E-01
S	125	146	2.95	8.58E-02	174	228	12.61***	3.84E-04
T	84	123	12.50***	4.08E-04	136	148	0.91	3.39E-01
V	147	168	2.73	9.88E-02	185	148	9.47**	2.09E-03
W	55	37	9.34**	2.24E-03	77	43	26.77***	2.30E-07
Y	84	87	0.11	7.40E-01	76	74	0.07	7.98E-01

Sum	2411	2411			2411	2411		

^a^χ^2^-numbers in italics indicate underrepresentation and numbers in bold overrepresentation compared to random distribution based on amino acid frequencies. The results of the χ^2 ^are shown with significance level: * *P *< 0.05; ** *P *< 0.01; *** *P *< 0.001.

**Table 3:** Spectrum of mutations appearing in α-helix, β-strand, turn and bend structures, expected values are calculated from mutated and mutant amino acid composition in the studied proteins^a^

Amino acid	Original residues	Expected residues	χ^2^	*P *value	Mutant residues	Expected residues	χ^2^	*P *value
A	148	136	1.07	3.00E-01	58	67	1.15	2.84E-01
C	54	64	1.43	2.32E-01	117	107	0.94	3.32E-01
D	81	79	0.06	8.06E-01	112	125	1.28	2.57E-01
E	96	91	0.29	5.91E-01	71	68	0.18	6.75E-01
F	53	53	0.00	9.91E-01	77	72	0.29	5.87E-01
G	220	198	2.48	1.15E-01	95	85	1.12	2.91E-01
H	63	70	0.69	4.05E-01	91	87	0.20	6.57E-01
I	83	80	0.14	7.05E-01	55	64	1.36	2.44E-01
K	43	45	0.09	7.61E-01	92	88	0.21	6.43E-01
L	188	186	0.03	8.70E-01	96	91	0.29	5.91E-01
M	65	63	0.08	7.74E-01	48	78	11.54***	6.79E-04
N	60	72	2.12	1.46E-01	61	60	0.04	8.47E-01
P	68	76	0.76	3.82E-01	170	156	1.25	2.63E-01
Q	45	50	0.47	4.91E-01	89	92	0.13	7.17E-01
R	299	280	1.31	2.53E-01	196	179	1.71	1.91E-01
S	78	101	5.05*	2.46E-02	144	140	0.12	7.31E-01
T	62	68	0.46	4.99E-01	119	109	0.85	3.57E-01
V	122	118	0.12	7.28E-01	138	149	0.78	3.77E-01
W	44	44	0.00	9.72E-01	56	62	0.57	4.51E-01
Y	67	68	0.00	9.46E-01	54	61	0.83	3.62E-01

Sum	1939	1939			1939	1939		

^a^χ^2^-numbers in italics indicate underrepresentation and numbers in bold overrepresentation compared to random distribution based on amino acid frequencies. The results of the χ^2 ^are shown with significance level: * *P *< 0.05; ** *P *< 0.01; *** *P *< 0.001.

First we investigated the statistical significance for mutated and mutant amino acids located in secondary structures compared to overall distribution. Then the patterns within each secondary structural element were revealed.

Arginine is clearly the most mutated residue type, as already indicated in previous studies [41-43]. The very high mutability of codons for arginine arises from CpG dinucleotides, which can spontaneously mutate by deamination either to TG or CA dinucleotides [44]. Arginine is coded by six codons, four of which have a CpG dinucleotide in the first and second codon position. In addition to the CpG dinucleotide we have also shown previously that the surrounding sequence context has an effect on mutability [42].

We calculated the relative mutability of all the amino acids as both mutant and mutated residues (Fig. 1). The residue that had the lowest ratio of observed vs. expected mutations (marked '1') was used as a reference in these calculations. This residue is lysine for mutated and alanine for mutant residues.

Among the original residues, arginine has clearly the highest relative mutability. The picture is completely different for the mutant residues, where the overall relative mutability among mutant residues is highest for cysteine and tryptophan. Table 2 shows that C, D, H, K, P and W are highly mutated residues. In the second place after arginine in mutated residues is cysteine. Glycine and tryptophan are also highly significantly overrepresented among mutated residues. Proline is the only amino acid forming a ring with the backbone and it has very rigid structure, which bends the main chain of the protein in a characteristic way. Proline is a known breaker of secondary structures [30]. K, E, Q, F and T are significantly underrepresented among mutated residues.

From Table 3 can be seen that the distribution of mutations in secondary structural elements is very close to that expected based on the mutation count in the whole proteins. Only S as mutated and M as mutant residue type are significantly underrepresented. Based on the amino acid distributions (Fig. 1) the composition of α-helices is close to the general distribution whereas β-structures and turns and bends have clearly different distribution. The amino acid compositions within secondary structures are very different. The share of α-helices is 36%, β-structures 21% and turns and bends 21% (remaining 21% is in areas not defined to any secondary structure type) of all the residues in the investigated structures. The secondary structures harbour 80% of all the disease-causing mutations. The amino acid distribution outside the regular secondary structural elements is very similar to whole protein except for high overrepresentation for P. The trends for mutated and mutant residues are similar to whole proteins. R has very high relative mutability for original residues (Fig. 1).

The only remarkable difference between Tables 2 and 4 (data calculated based on the amino acid distribution in secondary structures) is methionine, which is highly overrepresented in Table 2 for all the mutations as a mutant residue, but has no statistical correlation in the secondary structures (Table 4). A, L and I appear much less frequently amongst the replacing residues than expected. Our results are in line with those published previously for general mutation distribution [11,12].

**Table 4:** Spectrum of mutations appearing in α-helix, β-strand, turn and bend structures. Expected values are calculated from amino acid composition in secondary structural elements^a^

Amino acid	Original residues	Expected residues	χ^2^	*P *value	Mutant residues	Expected residues	χ^2^	*P *value
A	148	139	0,53	4.66E-01	58	119	31.05***	2.51E-08
C	54	32	15,50***	6.82E-05	117	69	32.93***	9.58E-09
D	81	96	2,34	1.26E-01	112	79	13.64***	2.21E-04
E	96	147	17,60***	2.71E-05	71	69	0.04	8.33E-01
F	53	87	13,51***	2.36E-04	77	79	0.06	8.10E-01
G	220	136	52,47***	4.48E-13	95	114	3.10	7.85E-02
H	63	48	5,06*	2.47E-02	91	79	1.78	1.83E-01
I	83	110	6,51**	1.07E-02	55	104	23.00***	1.62E-06
K	43	113	43,42***	4.37E-11	92	69	7.47**	6.26E-03
L	188	205	1,34	2.46E-01	96	163	27.69***	1.42E-07
M	65	49	5,02*	2.51E-02	48	45	0.27	6.02E-01
N	60	69	1,10	2.93E-01	61	79	4.16*	4.14E-02
P	68	67	0,02	8.98E-01	170	119	22.16***	2.51E-06
Q	45	82	16,87***	3.98E-05	89	69	5.63*	1.76E-02
R	299	119	272,51***	3.43E-61	196	168	4.60*	3.19E-02
S	78	104	6,60**	1.01E-02	144	183	8.32**	3.92E-03
T	62	92	10,03**	1.53E-03	119	119	0.00	9.79E-01
V	122	144	3,33	7.01E-02	138	119	3.13	7.67E-02
W	44	30	6,27**	1.23E-02	56	35	13.20***	2.81E-04
Y	67	70	0,15	6.95E-01	54	59	0.48	4.87E-01

Sum	1939	1939			1939	1939		

^a^χ^2^-numbers in italics indicate underrepresentation and numbers in bold overrepresentation compared to random distribution based on amino acid frequencies. The results of the χ^2 ^are shown with significance level: * *P *< 0.05; ** *P *< 0.01; *** *P *< 0.001.

### Mutational spectrum within α-helices, β-strands and turns and bends

The amino acid composition in α-helices is very similar to overall amino acid composition (Fig. 1). The only main difference is in the ratio of glycines and prolines, which are clearly depleted in the helices, whereas glycines, as expected, are especially strongly overrepresented in turns and bends. The most prominent residues in β-strands are aliphatic isoleucine, leucine and valine followed by alanine, phenylalanine and threonine. Aspartic acid is surprisingly frequent in turns and bends.

In the analysis of helix mutations in all secondary structures [see Additional file 1] glycine is very significantly underrepresented as mutated residue and also A and N have statistically significant values. M is the only significant mutant residue type. Correspondingly V, I and S have significant chi square values as mutated residues in β-strand, S, V and W as mutated residues [see Additional file 2]. In turns and bends the significantly mutated amino acids are S, V, A, D, I, K and L and as mutant residues E, M, and R [see Additional file 3].

We further investigated the mutations within these secondary structures. The distribution of disease-causing mutations in helical structures in the investigated 44 structures is shown in Table 5. The number of mutations in helices is altogether 928 (48%), whereas β-strand structures of extended strands and isolated β-bridges contain 550 (28%) missense mutations (Table 6) and hydrogen bonded turns and bends contain 461 (24%) mutations (Table 7). The results for original and mutant residues are clearly and significantly very different for the different structural elements. In helices, besides arginine and cysteine other residues are not very highly mutated. If only the secondary structures are taken into account, glycine appears to be the most mutated residue after arginine. This result is similar to a Steward et al. study that revealed that glycine was the second most frequently mutating amino acid in the OMIM disease database [9].

**Table 5:** Spectrum of mutations in α-helices^a^

Amino acid	Original residues	Expected residues	χ^2^	*P *value	Mutant residues	Expected residues	χ^2^	*P *value
A	89	83	0.45	5.03E-01	23	57	20.13***	7.25E-06
C	26	14	11.27***	7.88E-04	56	33	15.76***	7.18E-05
D	34	44	2.23	1.35E-01	59	38	11.78***	5.99E-04
E	61	96	12.71***	3.64E-04	26	33	1.54	2.15E-01
F	28	36	1.76	1.84E-01	37	38	0.02	8.87E-01
G	48	30	10.69***	1.08E-03	46	54	1.31	2.52E-01
H	23	21	0.13	7.19E-01	41	38	0.26	6.12E-01
I	41	51	1.96	1.61E-01	29	50	8.63**	3.30E-03
K	20	61	27.70***	1.41E-07	52	33	10.73***	1.05E-03
L	101	112	1.04	3.09E-01	49	78	10.86***	9.85E-04
M	41	29	5.11*	2.37E-02	24	21	0.34	5.59E-01
N	22	27	1.08	3.00E-01	29	38	2.08	1.49E-01
P	29	24	1.15	2.84E-01	90	57	19.38***	1.07E-05
Q	28	45	6.12*	1.34E-02	55	33	14.41***	1.47E-04
R	153	64	122.78***	1.56E-28	73	80	0.70	4.04E-01
S	36	46	2.22	1.36E-01	53	88	13.66***	2.19E-04
T	30	38	1.84	1.75E-01	61	57	0.31	5.79E-01
V	62	59	0.18	6.69E-01	69	57	2.61	1.06E-01
W	24	16	4.19*	4.06E-02	32	17	14.36***	1.51E-04
Y	32	33	0.01	9.29E-01	24	28	0.68	4.08E-01

Sum	928	928			928	928		

^a^χ^2^-numbers in italics indicate underrepresentation and numbers in bold overrepresentation compared to random distribution based on amino acid frequencies. The results of the χ^2 ^are shown with significance level: * *P *< 0.05; ** *P *< 0.01; *** *P *< 0.001.

**Table 6:** Spectrum of mutations in β-strands^a^

Amino acid	Original residues	Expected residues	χ^2^	*P *value	Mutant residues	Expected residues	χ^2^	*P *value
A	39	33	0.93	3.34E-01	14	34	11.49***	6.98E-04
C	19	12	4.58*	3.24E-02	39	20	19.08***	1.26E-05
D	17	15	0.22	6.38E-01	26	22	0.56	4.54E-01
E	21	25	0.64	4.23E-01	20	20	0.01	9.36E-01
F	15	36	12.66***	3.75E-04	24	22	0.11	7.43E-01
G	44	27	10.61***	1.13E-03	27	32	0.86	3.54E-01
H	23	13	8.33**	3.90E-03	32	22	4.06*	4.38E-02
I	34	49	4.80*	2.84E-02	16	29	6.15**	1.31E-02
K	13	23	4.60*	3.20E-02	23	20	0.57	4.49E-01
L	63	66	0.10	7.48E-01	27	46	8.05**	4.56E-03
M	14	13	0.03	8.53E-01	16	13	0.90	3.43E-01
N	19	14	1.67	1.97E-01	20	22	0.27	6.05E-01
P	15	11	1.08	2.99E-01	47	34	5.27*	2.16E-02
Q	10	16	2.24	1.34E-01	20	20	0.01	9.36E-01
R	77	29	80.03***	3.69E-19	60	48	3.17	7.50E-02
S	15	23	2.88	8.98E-02	55	52	0.18	6.68E-01
T	16	35	10.20***	1.41E-03	36	34	0.16	6.88E-01
V	53	71	4.44*	3.51E-02	27	34	1.32	2.50E-01
W	16	10	3.23	7.25E-02	8	10	0.34	5.61E-01
Y	27	27	0.00	9.60E-01	13	17	0.87	3.50E-01

Sum	550	550			550	550		

^a^χ^2^-numbers in italics indicate underrepresentation and numbers in bold overrepresentation compared to random distribution based on amino acid frequencies. The results of the χ^2 ^are shown with significance level: * *P *< 0.05; ** *P *< 0.01; *** *P *< 0.001.

**Table 7:** Spectrum of mutations in turns and bends^a^

Amino acid	Original residues	Expected residues	χ^2^	*P *value	Mutant residues	Expected residues	χ^2^	*P *value
A	20	24	0.79	3.73E-01	21	28	1.85	1.74E-01
C	9	7	0.97	3.26E-01	22	16	1.86	1.72E-01
D	30	35	0.70	4.04E-01	27	19	3.56	5.92E-02
E	14	27	6.28**	1.22E-02	25	16	4.43*	3.54E-02
F	10	16	2.13	1.45E-01	16	19	0.42	5.16E-01
G	128	72	43.23***	4.87E-11	22	27	0.94	3.32E-01
H	17	13	1.12	2.91E-01	18	19	0.04	8.51E-01
I	8	12	1.20	2.73E-01	10	25	8.75**	3.10E-03
K	10	28	11.80***	5.92E-04	17	16	0.02	8.95E-01
L	24	30	1.27	2.60E-01	20	39	9.12**	2.53E-03
M	10	8	0.67	4.13E-01	8	11	0.63	4.27E-01
N	19	26	1.72	1.90E-01	12	19	2.47	1.16E-01
P	24	29	1.02	3.12E-01	33	28	0.81	3.69E-01
Q	7	21	9.71**	1.84E-03	14	16	0.37	5.44E-01
R	69	26	70.17***	5.44E-17	63	40	13.25***	2.73E-04
S	27	34	1.27	2.59E-01	36	44	1.30	2.55E-01
T	16	20	0.68	4.09E-01	22	28	1.37	2.41E-01
V	7	17	6.05**	1.39E-02	42	28	6.72**	9.52E-03
W	4	5	0.07	7.92E-01	16	8	7.33**	6.78E-03
Y	8	11	1.02	3.13E-01	17	14	0.59	4.42E-01

Sum	461	461			461	461		

^a^χ^2^-numbers in italics indicate underrepresentation and numbers in bold overrepresentation compared to random distribution based on amino acid frequencies. The results of the χ^2 ^are shown with significance level: * *P *< 0.05; ** *P *< 0.01; *** *P *< 0.001.

In helices, lysine is a strongly underrepresented residue compared with the calculated expected value. P, C, Q, W and D show statistically significant overrepresentation when compared to expected values, while S and L are underrepresented among mutant amino acids. Relative mutability in α-helices indicates that arginine is 7.29 times more mutated than expected whereas glycine is 4.88 times so.

In β-structures, R is statistically very significantly overrepresented and G and H have weaker enrichment. T is the statistically least mutated residue in β-strands. Among mutant residues, C is highly overrepresented and A underrepresented. Otherwise the results are less biased than in helices.

R is also statistically the most overrepresented residue in turns and bends although the χ^2 ^score is smaller than for the other secondary structures. G is the only other statistically highly significantly overrepresented amino acid. G is the most flexible residue because it does not have a side chain. It often appears in tight turns where no other residue can replace it. K is highly underrepresented among the mutated residues. Interestingly, R is the most enriched amino acid among mutant residues.

The distributions of amino acid frequencies and relative mutabilities in the different structural elements (Fig. 1) are clearly very different. First of all, the values are higher for mutated residues than for mutant residues, indicating that the original residue in many instances is very important and substitutions to any other residue are not possible without detrimental effects. Arginine and glycine are among the most mutated residues in all secondary structures whereas the other amino acids have large variations between the secondary structure types. The effect of arginine can also be seen in the data for mutant residues. Point mutations in arginine lead mostly to cysteine and glutamine, which have relatively high mutability values. The mutant residues in turns and bends have a more even distribution than the other two elements because practically any mutation to key residues in a tight hairpin turn leads either to the loss of hydrogen bonds or does not allow tight turn formation and leads to consequent structural alterations.

The data for mutations outside regular elements (Table 8) indicate that R, H and Y are significantly overrepresented and Q, K and E underrepresented among mutated residues while N is the only residue type that is significantly overrepresented.

**Table 8:** Mutated and mutant residues not in secondary structures^a^

Amino acid group	Original residues	Expected residues	χ^2^	P value	Mutant residues	Expected residues	χ^2^	P value
A	18	22	0.84	3.58E-01	21	29	2.16	1.42E-01
C	12	7	4.51	3.37E-02	25	17	3.93*	4.73E-02
D	46	34	4.20	4.04E-02	17	19	0.27	6.06E-01
E	10	22	6.53**	1.06E-02	17	17	0.00	9.72E-01
F	15	17	0.17	6.83E-01	13	19	2.04	1.53E-01
G	35	32	0.37	5.40E-01	26	28	0.10	7.48E-01
H	27	14	13.05***	3.04E-04	24	19	1.16	2.81E-01
I	19	17	0.23	6.32E-01	16	25	3.41	6.48E-02
K	11	27	9.15**	2.49E-03	13	17	0.88	3.47E-01
L	29	36	1.36	2.44E-01	43	40	0.27	6.04E-01
M	6	9	0.93	3.36E-01	13	11	0.43	5.11E-01
N	10	23	6.96**	8.34E-03	30	19	5.98**	1.45E-02
P	61	52	1.44	2.29E-01	26	29	0.29	5.90E-01
Q	3	20	13.97***	1.86E-04	17	17	0.00	9.72E-01
R	53	23	37.42***	9.51E-10	49	41	1.59	2.08E-01
S	31	40	2.13	1.45E-01	47	45	0.13	7.14E-01
T	24	30	1.25	2.63E-01	22	29	1.65	1.99E-01
V	25	25	0.00	9.44E-01	25	29	0.53	4.68E-01
W	8	6	0.41	5.22E-01	11	8	0.78	3.76E-01
Y	29	17	8.77**	3.06E-03	17	14	0.45	5.02E-01

Sum	472	472			472	472		

^a^χ^2^-numbers in italics indicate underrepresentation and numbers in bold overrepresentation compared to random distribution based on amino acid frequencies. The results of the χ^2 ^are shown with significance level: * *P *< 0.05; ** *P *< 0.01; *** *P *< 0.001.

### Mutational spectrum in amino acid groups within structural elements

Amino acids can be grouped based on their physicochemical nature. The reason for performing an analysis with residue categories is that in many sites the specific amino acid is not very important but the suitable properties it provides is. This can be seen e.g. in multiple sequence alignments of protein families. We used the following six groups: hydrophobic (V, I, L, F, M, W, Y, C), positively charged (R, K, H), negatively charged (D and E), conformational (G and P), polar (N, Q, S) and (A and T) [45]. These groups follow the amino acid substitution matrices used for sequence alignments and database searches. The results for amino acid frequencies in the secondary structure types are in Tables 9, 10, 11. Different groups are over- or underrepresented in different structure types. Negatively charged residues, due to arginine, are overrepresented in all three tables. Conformational residues are also overrepresented in all three secondary structure classes, but with varying χ^2^-values. Positively charged amino acids are significantly underrepresented in helices and overrepresented in turns and bends.

**Table 9:** Spectrum of mutations in α-helices in amino acid groups^a^

Amino acid group	Original residues	Expected residues	χ^2^	*P *value	Mutant residues	Expected residues	χ^2^	*P *value
Hydrophobic	355	348	0.13	7.19E-01	320	322	0.01	9.13E-01
Positively charged	95	140	14.36***	1.51E-04	85	71	2.75	9.71E-02
Negatively charged	196	147	16.56***	4.71E-05	166	152	1.39	2.39E-01
Conformational	77	54	9.96**	1.60E-03	136	111	5.50*	1.90E-02
Polar	86	118	8.70**	3.17E-03	137	159	2.94	8.62E-02
A+T	119	121	0.04	8.34E-01	84	114	7.73**	5.44E-03

Sum	928	928			928	928		

^a^χ^2^-numbers in italics indicate underrepresentation and numbers in bold overrepresentation compared to random distribution based on amino acid frequencies. The results of the χ^2 ^are shown with significance level: * *P *< 0.05; ** *P *< 0.01; *** *P *< 0.001.

**Table 10:** Spectrum of mutations in β-strands in amino acid groups^a^

Amino acid group	Original residues	Expected residues	χ^2^	*P *value	Mutant residues	Expected residues	χ^2^	*P *value
Hydrophobic	241	285	6.72**	9.54E-03	170	191	2.27	1.32E-01
Positively charged	38	40	0.12	7.31E-01	46	42	0.36	5.47E-01
Negatively charged	113	65	35.48***	2.58E-09	115	90	7.07**	7.82E-03
Conformational	59	39	10.86***	9.81E-04	74	66	0.98	3.21E-01
Polar	44	53	1.62	2.03E-01	95	94	0.01	9.18E-01
A+T	55	68	2.58	1.08E-01	50	67	4.47*	3.45E-02

Sum	550	550			550	550		

^a^χ^2^-numbers in italics indicate underrepresentation and numbers in bold overrepresentation compared to random distribution based on amino acid frequencies. The results of the χ^2 ^are shown with significance level: * *P *< 0.05; ** *P *< 0.01; *** *P *< 0.001.

**Table 11:** Spectrum of mutations in turns and bends in amino acid groups^a^

Amino acid group	Original residues	Expected residues	χ^2^	*P *value	Mutant residues	Expected residues	χ^2^	*P *value
Hydrophobic	80	105	6.02**	1.42E-02	151	160	0.50	4.80E-01
Positively charged	44	62	5.21*	2.24E-02	52	35	7.92**	4.88E-03
Negatively charged	96	68	11.94***	5.48E-04	98	75	6.87**	8.78E-03
Conformational	152	102	24.95***	5.90E-07	55	55	0.00	9.71E-01
Polar	53	81	9.44**	2.12E-03	62	79	3.58	5.85E-02
A+T	36	44	1.48	2.24E-01	43	56	3.20	7.34E-02

Sum	461	461			461	461		

^a^χ^2^-numbers in italics indicate underrepresentation and numbers in bold overrepresentation compared to random distribution based on amino acid frequencies. The results of the χ^2 ^are shown with significance level: * *P *< 0.05; ** *P *< 0.01; *** *P *< 0.001.

Mutations to polar residues are significantly depleted in α-helices and especially so in turns and bends. The number of significant observations is lower in mutant residues. Negatively charged amino acids are overrepresented in both β-structures and in bends and turns, although the enrichment is relatively weak. Alanine and threonine are underrepresented in both helices and β-strands. These results likely reflect the importance of the original residue. Many disease-causing mutations affect the same positions indicating that the site is structurally or functionally important, where any mutation disrupts the protein activity.

### Changes in amino acid volume and charge due to mutations

**Figure 2:**
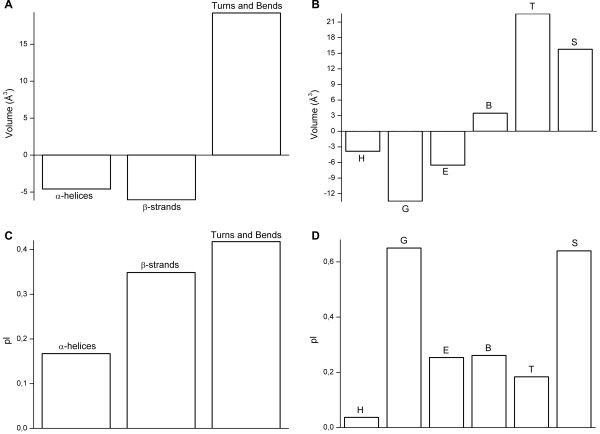
**Changes to properties of amino acids caused by mutations**. Average changes per residue in residue volumes when comparing original amino acid and mutated amino acid in A) α-helices, β-strands, turns and bends and B) separately in α-helices (H), 3_10_-helices (G), extended strands (E), isolated β-bridges (B), turns (T) and bends (S). C) Average changes per residue in charges when comparing original amino acid and mutated amino acid in α-helices, β-strands, turns and bends and D) separately in α-helices, 3_10_-helices, extended strands, isolated β-bridges, turns and bends.

**Figure 3:**
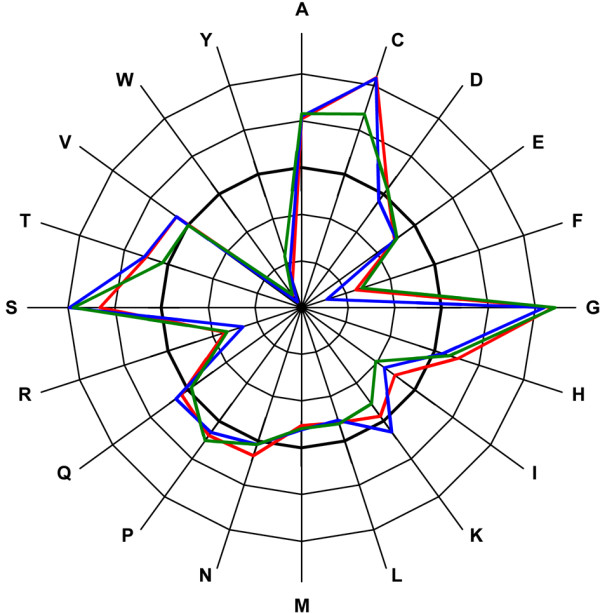
**Changes in mutations to residue volumes**. Average changes in mutations to residue volumes in α-helices (red), β-strands (blue), turns and bends (green). The thick line indicates the original amino acid volumes. Outer rings indicate addition in volume and inner rings reduction, in steps of 35 Å^3^.

Next we examined the differences in volume and charge between the original residue and the replacing mutant residue (Fig. 2). The replacing residues within α-helices and β-strand structures are generally physically smaller than the original residues while in turns and bends the volume is remarkably increased. In 3_10_-helices the replacing residue is on average 13.5 Å^3 ^smaller than the original residue and in α-helices almost 4 Å^3 ^smaller. Mutations to residues in β-bridges increase the volume, while mutations in β-strands on average reduce the volume of the amino acid. The increase in volume in turns is 22.6 Å^3 ^and in bends 15.8 Å^3^, thus on average the replacing residue is much larger than the original residue. In turns and bends, glycines form the biggest group among mutated native amino acids. Because glycine is the smallest amino acid, all mutations in G increase the volume of the protein. Differences at the amino acid level show that there are clear peaks in some residues, and that the changes in all secondary structure types are very similar (Fig. 3). Glycine (68 Å^3^) is replaced by residues that are on average 85 Å^3 ^larger in turns and bends, 83 Å^3 ^larger in α-helices and 78 Å^3 ^larger in β-strands. The largest residues are W, Y, F and R. The largest amino acid, tryptophan (237 Å^3^) is replaced by residues whose volume is on average 100 Å^3 ^smaller in β-strands and 94 Å^3 ^and 93 Å^3 ^smaller in α-helices and turns and bends, respectively. Being a bulky residue, tryptophan is very rare in turns and bends. In our dataset W accounted for 1% of all residues in turns and bends. There was just one mutated W in bends and three in turns.

**Figure 4:**
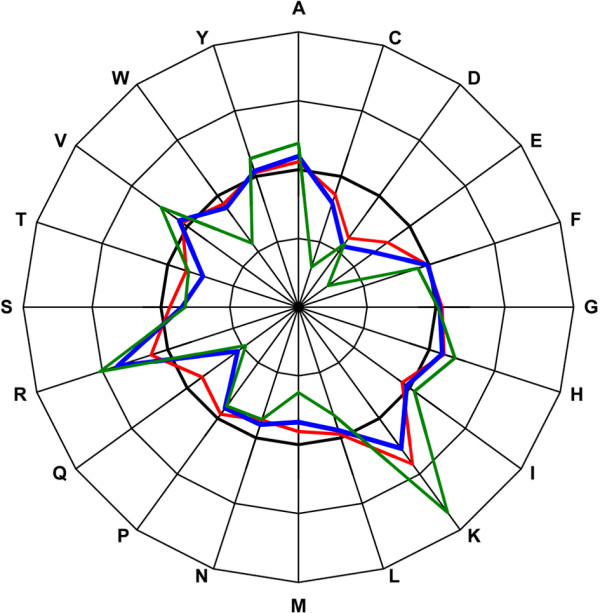
**Changes in mutations to residue charges**. Average changes in mutations to residue charges in α-helices (red), β-strands (blue), turns and bends (green). The thick line indicates the original amino acid charges. Outer rings indicate higher charge values and inner rings lower, in steps of 1.25 pI.

Average changes in isoelectric point (IEP) are similar for the main secondary structure types (Fig. 2C). In all the structures the IEP is increased on average compared to the normal structure. There are differences in the extent of the change, the largest changes are in turns and bends – on average close to 0.8 pI. The reason for the dramatic average increase in pI values is the introduction of prolines and lysines that have relatively high pI values. Arginine has the highest pI value of all amino acids, but in turns and bends there is almost equally high enrichments of arginines both in mutated and mutant residues, so arginines do not affect IEP crucially. Figure 2D also shows in detail the changes in different helices and β-structures. The change is much larger in 3_10_-helices than in α-helices. A similar situation is seen for bends in which mutations lead to clearly larger changes. Differences at the amino acid level show that changes in charge in α-helices and β-strand structures are minor but in turns and bends there are clear peaks in some of the residues. C and E are replaced by residues with lower pI values and basic K and R by residues with higher pI values (Fig. 4).

### The role of contact energies

**Figure 5:**
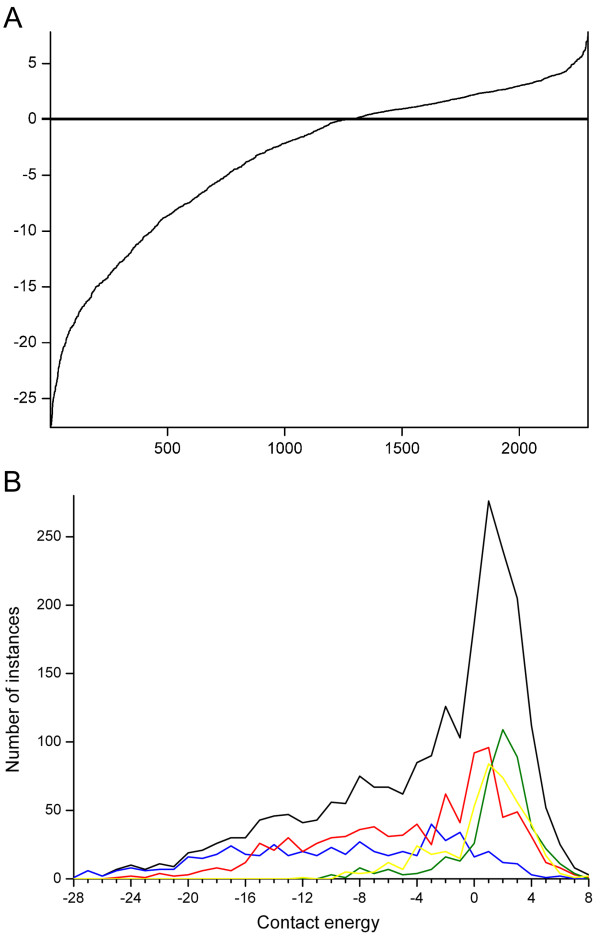
**Distribution of contact energies for mutated amino acids**. The energies were calculated with the RankViaContact program. A) Data for all mutations, and B) distribution in secondary structural elements α-helices (red), β-strands (blue), turns and bends (green), outside secondary structures (yellow), and whole proteins (black).

We used RankViaContact [46] to calculate residue contact energies for all proteins in the dataset based on three-dimensional structures. The contact energies of the mutated residues range from very strong -27.6 to 7.8. The mutations are ranked based on their calculated contact energies. A slight majority (55%) of the mutations have strong or very strong contact energies. Most residues with strong contact energies are important for the stability of the protein structure. Many of the disease-related mutations are thus located in crucial structural sites in which alterations are deleterious. In figure 5A the count of contact energies of the mutated residues has been calculated in all proteins and separately for secondary structures. In β-strand the distribution of contact energies are even, but in other structures the distribution is biased towards weak positive contact energies.

Figure 5B shows the contact energy distribution for mutations in proteins and secondary structure types as well as outside the secondary structural elements. The curves have quite similar overall shapes although the location of the peak for the maximal occurrence varies. Of note is also that the mutation positions in turns and bends and outside secondary structures do not appear in sites of very strong contacts. Mutation sites in β-structures have very even distribution throughout the contact energy range.

**Figure 6:**
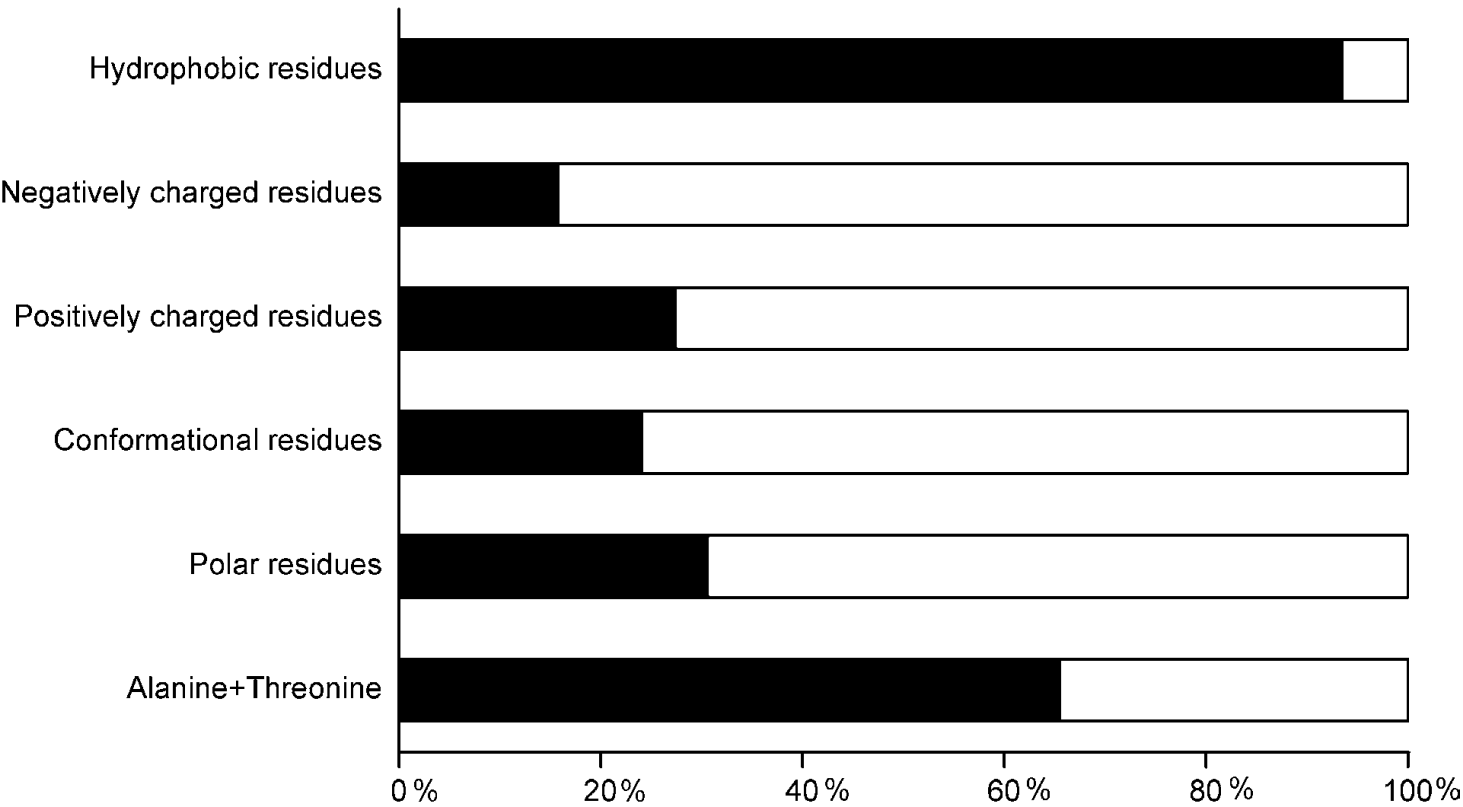
**Mutant residues with strong and weak contact energies**. The percentage of mutant residues with strong (black) and weak (white) contact energies in physicochemical amino acid groups.

We organized the residues into six groups based on their physicochemical properties and calculated the percentages of strong and weak contact energies in the groups (Fig. 6). Among hydrophobic residues more than 90% of the original amino acids have strong contact energies. Polar, conformational, positively and negatively charged mutated residues have for the most part weak contact energies (70–85%). A and T residues have mainly strong contact energies. These results indicate the importance of the residue type for interactions – whether hydrophobic, van der Waals or electronic interactions. Charged residues often form salt bridges, while hydrophobic and aliphatic amino acids are involved in weaker interactions. A large proportion of mutated residues are forming interactions which are essential for the protein and its structure. Structural alterations are the most common consequence of disease-causing mutations [18].

## Conclusion

Germline substitutions leading to the replacement of a native residue can result in either benign effects (e.g. polymorphisms) or in genetic disease. It has been shown that most positions in proteins can be altered without serious effects on protein structure or function [47]. On the other hand, the majority of disease-causing mutations have structural effects [14]. Therefore, mutations that are phenotypic for disease indicate the importance for the specific location. Knowledge of the molecular basis of mutations is important and can be used in several ways.

Mutation spectra are clearly different for the different structural elements. The most prominent feature for all the elements is the strong overrepresentation of arginine as mutated residue. Large changes in the properties of mutant amino acids, in volume or charge, are disease-related, but there are large differences for the structural elements. Contact energy distributions of mutated residues are surprisingly similar except for β structures. About half of the mutation sites are involved in strong or very strong amino acid interactions. In conclusion, there are many and strong trends in mutant and mutated residues. The trends are statistically significantly different for the different secondary structures.

Previously, mutation statistics have been studied at a general level [8,12,48] as well as a structural level [9,12], but detailed analysis of the spectrum and effects of mutations within secondary structural elements has been missing. In the study of Ferrer-Costa et al. [12] the dataset consisted of 1169 disease-associated single amino acid polymorphisms (daSAPs) distributed over 73 proteins with structure information from the PDB. The study combines information from all secondary structure elements without element specific data. In the study of Steward and co-workers [9] 63 proteins contained 1292 disease-associated sites. They did not analyse secondary structures separately. Our study involved 1939 missense mutations in secondary structures and 2411 mutations altogether. We localized the majority of missense mutations to α-helices. The biggest group of daSAPs is found in coils, and helices contain 36.7% of all daSAPs. Ferrer-Costa et al. also included volume comparisons in their study. In contrast to our work, the size changes to daSAPs were calculated for the whole dataset. We calculated the changes for each secondary structure type separately, also at amino acid level. Arginine is the most commonly mutated residue. The result is identical to Steward et al [9]. Wang and Moult [18] examined 262 missense mutations in 23 proteins and concluded that 80% of mutations destabilize the protein structure relative to the folded state based on changes in hydrophobic burial, backbone strain, overpacking, and electrostatic interactions. In several other studies, structural properties have been combined with a sequence profile for the mutated position [21,23,49], which reflects the occurrence of other amino acid types at corresponding sites in homologous proteins.

Vitkup et al. [11] investigated in total, 4236 mutations from 436 genes and concluded that mutations at arginine and glycine residues are together responsible for about 30% of genetic diseases. This result is similar to ours. In our dataset, 25% of all missense mutations occur in R and G, and 23% are present in the examined secondary structures. Vitkup et al. also found that random mutations at tryptophan and cysteine have the highest probability of causing disease, which is in line with our results. The overall relative mutability to mutant residues is 3.46 for C and 3.31 for W compared to alanine which has the lowest ratio of observed vs. expected mutations and is considered as 1.

To correlate the mutated and mutant types to protein structures it is important to know at which secondary structural element the alteration appears. Protein function and interactions require both stability and specificity. Proteins fold according to the minimum free energy. In contrast, they organize themselves to recognize a transition state or a ligand [50]. Amino acids and mutations have very distinct differences in the frequencies in the secondary structural elements. Multiple sequence alignments can be used to investigate allowed substitutions in protein families. Our analysis revealed mutation types which are most likely deleterious. This information could also be used for the development and optimization of amino acid comparison tables for individual secondary structural elements. Our data could also add to the reliability of the predictions of mutation effects.

## Methods

We investigated proteins for which numerous disease-causing missense mutations were available along with an experimentally defined three-dimensional structure. Mutation data was collected from BTKbase [51-53] for Btk mutations, CD40Lbase [54-56] for CD40L mutations and from Human Gene Mutation Database (HGMD) [6] for the remainder. The publicly available mutations are in the supplementary table 4 [see Additional file 4], full list is available from authors by request. The DSSP program [57] was used to identify secondary structures for three helix types, two extended β-strand types, turns and bends, based on the stereochemistry of the protein structure. The DSSP program was used to systematically assign secondary structures for each residue in the three-dimensional structures obtained from the Protein Data Bank (PDB) [58]. A Perl script was written to connect mutation information to protein structure data (dssp files).

If more than one structure existed for a protein or a protein domain, the one with the highest resolution and the longest chain length was chosen. All data was stored in a mySQL database. Altogether we investigated 44 proteins with a total of 12540 residues, of which 9878 were in the examined secondary structures. The proteins contained 2411 different disease-causing mutations, 1939 present in secondary structures.

The RankViaContact service was used to calculate residue-residue contact energies based on a coarse-grained model [46]. The energy parameters used for residue-residue contacts [59] were derived considering the secondary structural environments. The contact energies were estimated for all the missense mutations located in the secondary structures.

Mutation statistics were analysed by comparing the frequencies of the obtained mutations with the expected values. Expected values for mutated residues within α-helices, β-strands and turns and bends were calculated using the distribution of all amino acids in respective secondary structure. In the case of the mutant amino acids within different secondary structures, the expected values were calculated from codon diversity by taking into account all possible amino acid substitutions. In order to reveal how the mutation distribution in secondary structures compares to the overall distribution of mutations in the dataset, the expectation values for mutated and mutant residues were calculated based on all the mutations in secondary structures.

The χ^2 ^test was used to determine the significance of the results. Chi square values were calculated using the following formula:

χ2=Σ(fo−fe)2,fe
 MathType@MTEF@5@5@+=feaafiart1ev1aaatCvAUfKttLearuWrP9MDH5MBPbIqV92AaeXatLxBI9gBaebbnrfifHhDYfgasaacH8akY=wiFfYdH8Gipec8Eeeu0xXdbba9frFj0=OqFfea0dXdd9vqai=hGuQ8kuc9pgc9s8qqaq=dirpe0xb9q8qiLsFr0=vr0=vr0dc8meaabaqaciaacaGaaeqabaqabeGadaaakeaaiiGacqWFhpWydaahaaWcbeqaaiabikdaYaaakiabg2da9iabfo6atnaalaaabaGaeiikaGIaemOzay2aaSbaaSqaaiabd+gaVbqabaGccqGHsislcqWGMbGzdaWgaaWcbaGaemyzaugabeaakiabcMcaPmaaCaaaleqabaGaeGOmaiJaeiilaWcaaaGcbaGaemOzay2aaSbaaSqaaiabdwgaLbqabaaaaaaa@3F79@

where *f*_*o *_is the observed frequency and *f*_*e *_is the expected frequency for an amino acid. *P*-values and 95% confidence intervals were estimated in one-tailed fashion. Relative mutability was calculated using the formula:

Rm(N)=(Nobs⋅N′exp)(N′obs⋅Nexp),
 MathType@MTEF@5@5@+=feaafiart1ev1aaatCvAUfKttLearuWrP9MDH5MBPbIqV92AaeXatLxBI9gBaebbnrfifHhDYfgasaacH8akY=wiFfYdH8Gipec8Eeeu0xXdbba9frFj0=OqFfea0dXdd9vqai=hGuQ8kuc9pgc9s8qqaq=dirpe0xb9q8qiLsFr0=vr0=vr0dc8meaabaqaciaacaGaaeqabaqabeGadaaakeaacqWGsbGucqWGTbqBcqGGOaakcqWGobGtcqGGPaqkcqGH9aqpdaWcaaqaaiabcIcaOiabd6eaonaaBaaaleaacqWGVbWBcqWGIbGycqWGZbWCaeqaaOGaeyyXICTafmOta4KbauaadaWgaaWcbaGaemyzauMaemiEaGNaemiCaahabeaakiabcMcaPaqaaiabcIcaOiqbd6eaozaafaWaaSbaaSqaaiabd+gaVjabdkgaIjabdohaZbqabaGccqGHflY1cqWGobGtdaWgaaWcbaGaemyzauMaemiEaGNaemiCaahabeaakiabcMcaPaaacqGGSaalaaa@5235@

where *N' *is the least mutated residue type that was obtained by calculating the ratio between observed and expected value. *N *represents the number of mutated original or mutant residues for an amino acid type. The relative mutability was calculated for each residue type in all the investigated secondary structural elements.

In order to calculate changes in the isoelectric point we used the Emboss iep program [60] to predict IEPs for native and mutant proteins. The average change in IEP in each secondary structure type was calculated. The changes in residue volumes in different secondary structure types were obtained by using residue volumes [61]. The results were weighted by the amount of individual mutations and averaged by the number of mutant residues in respective secondary structures.

## Authors' contributions

SK and MV designed the study together. SK collected data, formed the database and performed the statistical analysis. All authors read and approved the final manuscript.

## Supplementary Material

Additional file 1 Spectrum of mutations appearing in α-helix. Expected values are calculated from mutated and mutant amino acid composition in the studied proteins. Click here for file

Additional file 2 Spectrum of mutations appearing in β-strand. Expected values are calculated from mutated and mutant amino acid composition in the studied proteins. Click here for file

Additional file 3 Spectrum of mutations appearing in turn and bend structures. Expected values are calculated from mutated and mutant amino acid composition in the studied proteins. Click here for file

Additional file 4 The publicly available mutations. List of analysed mutations obtained from publicly available databases, BTKbase, CD40Lbase and SwissProt Click here for file
