# Supplementary material for: Spectrum of disease-causing mutations in protein secondary structures
Source: BMC Struct Biol. 2007 Aug 29;7:56. doi: 10.1186/1472-6807-7-56 (PMC1995201; doi:10.1186/1472-6807-7-56)
Supplement: Additional file 1 — Spectrum of mutations appearing in α-helix. Expected values are calculated from mutated and mutant amino acid composition in the studied proteins. [file 1472-6807-7-56-S1.doc]

Supplementary table 1: Spectrum of mutations appearing in α-helix. Expected values are calculated from mutated and mutant amino acid composition in the studied proteins^a^

| Amino acid  group | Original  residues | Expected  residues | χ2 | P value | Mutant  residues | Expected  residues | χ2 | P value |
| --- | --- | --- | --- | --- | --- | --- | --- | --- |
| A | 89 | 65 | **8.82**** | 2.98E-03 | 23 | 32 | 2.51 | 1.13E-01 |
| C | 26 | 30 | 0.64 | 4.24E-01 | 56 | 51 | 0.45 | 5.02E-01 |
| D | 34 | 38 | 0.37 | 5.45E-01 | 59 | 60 | 0.01 | 9.32E-01 |
| E | 61 | 43 | **7.05**** | 7.94E-03 | 26 | 32 | 1.24 | 2.65E-01 |
| F | 28 | 25 | 0.27 | 6.06E-01 | 37 | 35 | 0.16 | 6.89E-01 |
| G | 48 | 95 | *23.02**** | 1.60E-06 | 46 | 41 | 0.66 | 4.16E-01 |
| H | 23 | 33 | 3.28 | 7.00E-02 | 41 | 42 | 0.01 | 9.30E-01 |
| I | 41 | 38 | 0.22 | 6.39E-01 | 29 | 31 | 0.10 | 7.47E-01 |
| K | 20 | 22 | 0.11 | 7.38E-01 | 52 | 42 | 2.41 | 1.21E-01 |
| L | 101 | 89 | 1.64 | 2.00E-01 | 49 | 43 | 0.70 | 4.04E-01 |
| M | 41 | 30 | 4.01 | 4.51E-02 | 24 | 37 | *4.76** | 2.91E-02 |
| N | 22 | 35 | *4.61** | 3.17E-02 | 29 | 28 | 0.01 | 9.23E-01 |
| P | 29 | 36 | 1.43 | 2.33E-01 | 90 | 75 | 3.15 | 7.61E-02 |
| Q | 28 | 24 | 0.72 | 3.97E-01 | 55 | 44 | 2.60 | 1.07E-01 |
| R | 153 | 134 | 2.71 | 9.97E-02 | 73 | 85 | 1.81 | 1.78E-01 |
| S | 36 | 48 | 3.05 | 8.08E-02 | 53 | 67 | 2.92 | 8.77E-02 |
| T | 30 | 32 | 0.17 | 6.82E-01 | 61 | 52 | 1.43 | 2.32E-01 |
| V | 62 | 57 | 0.52 | 4.71E-01 | 69 | 71 | 0.07 | 7.94E-01 |
| W | 24 | 21 | 0.38 | 5.38E-01 | 32 | 30 | 0.19 | 6.64E-01 |
| Y | 32 | 32 | 0.00 | 9.53E-01 | 24 | 29 | 0.94 | 3.31E-01 |
| Sum | 928 | 928 |  |  | 928 | 928 |  |  |

^a^χ^2^-numbers in italics indicate underrepresentation and numbers in bold overrepresentation compared to random distribution based on amino acid frequencies. The results of the χ^2^ are shown with significance level: * *P* < 0.05; ** *P* < 0.01; *** *P* < 0.001.
