# Supplementary material for: Spectrum of disease-causing mutations in protein secondary structures
Source: BMC Struct Biol. 2007 Aug 29;7:56. doi: 10.1186/1472-6807-7-56 (PMC1995201; doi:10.1186/1472-6807-7-56)
Supplement: Additional file 2 — Spectrum of mutations appearing in β-strand. Expected values are calculated from mutated and mutant amino acid composition in the studied proteins. [file 1472-6807-7-56-S2.doc]

Supplementary table 2: Spectrum of mutations appearing in β-strand. Expected values are calculated from mutated and mutant amino acid composition in the studied proteinsa

| Amino acid  group | Original  residues | Expected  residues | χ2 | P value | Mutant  residues | Expected  residues | χ2 | P value |
| --- | --- | --- | --- | --- | --- | --- | --- | --- |
| A | 39 | 39 | 0.01 | 9.43E-01 | 14 | 19 | 1.29 | 2.57E-01 |
| C | 19 | 18 | 0.05 | 8.18E-01 | 39 | 30 | 2.47 | 1.16E-01 |
| D | 17 | 22 | 1.28 | 2.57E-01 | 26 | 35 | 2.48 | 1.16E-01 |
| E | 21 | 26 | 0.89 | 3.47E-01 | 20 | 19 | 0.04 | 8.48E-01 |
| F | 15 | 15 | 0.00 | 9.88E-01 | 24 | 21 | 0.59 | 4.44E-01 |
| G | 44 | 56 | 2.62 | 1.06E-01 | 27 | 24 | 0.33 | 5.66E-01 |
| H | 23 | 20 | 0.50 | 4.79E-01 | 32 | 25 | 2.20 | 1.38E-01 |
| I | 34 | 23 | **5.77*** | 1.63E-02 | 16 | 18 | 0.28 | 5.98E-01 |
| K | 13 | 13 | 0.00 | 9.50E-01 | 23 | 25 | 0.14 | 7.08E-01 |
| L | 63 | 53 | 2.01 | 1.56E-01 | 27 | 26 | 0.06 | 8.10E-01 |
| M | 14 | 18 | 0.81 | 3.68E-01 | 16 | 22 | 1.70 | 1.93E-01 |
| N | 19 | 21 | 0.11 | 7.35E-01 | 20 | 17 | 0.58 | 4.48E-01 |
| P | 15 | 21 | 1.94 | 1.64E-01 | 47 | 44 | 0.17 | 6.80E-01 |
| Q | 10 | 14 | 1.21 | 2.71E-01 | 20 | 26 | 1.48 | 2.24E-01 |
| R | 77 | 79 | 0.07 | 7.89E-01 | 60 | 51 | 1.73 | 1.89E-01 |
| S | 15 | 29 | *6.41*** | 1.14E-02 | 55 | 40 | **5.90*** | 1.51E-02 |
| T | 16 | 19 | 0.52 | 4.70E-01 | 36 | 31 | 0.80 | 3.72E-01 |
| V | 53 | 34 | **11.30***** | 7.75E-04 | 27 | 42 | *5.48** | 1.93E-02 |
| W | 16 | 13 | 0.95 | 3.30E-01 | 8 | 18 | *5.21** | 2.25E-02 |
| Y | 27 | 19 | 3.21 | 7.34E-02 | 13 | 17 | 1.09 | 2.98E-01 |
| Sum | 550 | 550 |  |  | 550 | 550 |  |  |

^a^χ^2^-numbers in italics indicate underrepresentation and numbers in bold overrepresentation compared to random distribution based on amino acid frequencies. The results of the χ^2^ are shown with significance level: * *P* < 0.05; ** *P* < 0.01; *** *P* < 0.001.
