# Supplementary material for: Spectrum of disease-causing mutations in protein secondary structures
Source: BMC Struct Biol. 2007 Aug 29;7:56. doi: 10.1186/1472-6807-7-56 (PMC1995201; doi:10.1186/1472-6807-7-56)
Supplement: Additional file 3 — Spectrum of mutations appearing in turn and bend structures. Expected values are calculated from mutated and mutant amino acid composition in the studied proteins. [file 1472-6807-7-56-S3.doc]

Supplementary table 3: Spectrum of mutations appearing in turn and bend structures. Expected values are calculated from mutated and mutant amino acid composition in the studied proteinsa

| Amino acid  group | Original  residues | Expected  residues | *χ2* | P value | Mutant  residues | Expected  residues | χ2 | P value |
| --- | --- | --- | --- | --- | --- | --- | --- | --- |
| A | 20 | 32 | *4.69** | 3.03E-02 | 21 | 16 | 1.66 | 1.98E-01 |
| C | 9 | 15 | 2.47 | 1.16E-01 | 22 | 25 | 0.46 | 4.96E-01 |
| D | 30 | 19 | **6.77**** | 9.28E-03 | 27 | 30 | 0.23 | 6.28E-01 |
| E | 14 | 22 | 2.68 | 1.02E-01 | 25 | 16 | **4.97*** | 2.57E-02 |
| F | 10 | 13 | 0.54 | 4.61E-01 | 16 | 17 | 0.08 | 7.71E-01 |
| G | 128 | 47 | **139.36***** | 3.68E-32 | 22 | 20 | 0.15 | 7.00E-01 |
| H | 17 | 17 | 0.01 | 9.29E-01 | 18 | 21 | 0.34 | 5.60E-01 |
| I | 8 | 19 | *6.31*** | 1.20E-02 | 10 | 15 | 1.83 | 1.76E-01 |
| K | 10 | 11 | 0.05 | 8.29E-01 | 17 | 21 | 0.71 | 4.00E-01 |
| L | 24 | 44 | *9.21*** | 2.41E-03 | 20 | 22 | 0.12 | 7.30E-01 |
| M | 10 | 15 | 1.62 | 2.03E-01 | 8 | 19 | *6.00*** | 1.43E-02 |
| N | 19 | 17 | 0.19 | 6.66E-01 | 12 | 14 | 0.33 | 5.68E-01 |
| P | 24 | 18 | 2.02 | 1.55E-01 | 33 | 37 | 0.45 | 5.01E-01 |
| Q | 7 | 12 | 1.99 | 1.59E-01 | 14 | 22 | 2.90 | 8.84E-02 |
| R | 69 | 67 | 0.09 | 7.63E-01 | 63 | 42 | **9.95**** | 1.61E-03 |
| S | 27 | 24 | 0.40 | 5.26E-01 | 36 | 33 | 0.22 | 6.36E-01 |
| T | 16 | 16 | 0.00 | 9.88E-01 | 22 | 26 | 0.62 | 4.32E-01 |
| V | 7 | 28 | *15.85**** | 6.85E-05 | 42 | 35 | 1.24 | 2.65E-01 |
| W | 4 | 11 | 4.04 | 4.45E-02 | 16 | 15 | 0.11 | 7.39E-01 |
| Y | 8 | 16 | 4.05 | 4.43E-02 | 17 | 15 | 0.42 | 5.17E-01 |
| Sum | 461 | 461 |  |  | 461 | 461 |  |  |

^a^χ^2^-numbers in italics indicate underrepresentation and numbers in bold overrepresentation compared to random distribution based on amino acid frequencies. The results of the χ^2^ are shown with significance level: * *P* < 0.05; ** *P* < 0.01; *** *P* < 0.001.
