# Supplementary material for: Spectrum of disease-causing mutations in protein secondary structures
Source: BMC Struct Biol. 2007 Aug 29;7:56. doi: 10.1186/1472-6807-7-56 (PMC1995201; doi:10.1186/1472-6807-7-56)
Supplement: Additional file 4 — The publicly available mutations. List of analysed mutations obtained from publicly available databases, BTKbase, CD40Lbase and SwissProt [file 1472-6807-7-56-S4.doc]

Supplementary table 4: List of analysed mutations obtained from publicly available databases, BTKbase, CD40Lbase and SwissProt

| Name | Accession number | Original residue | Mutant residue |
| --- | --- | --- | --- |
| ABO | P16442 | P | S |
| ABO | P16442 | F | I |
| ABO | P16442 | V | M |
| AGXT | P21549 | P | L |
| AGXT | P21549 | G | R |
| AGXT | P21549 | G | V |
| AGXT | P21549 | G | E |
| AGXT | P21549 | G | R |
| AGXT | P21549 | F | I |
| AGXT | P21549 | G | R |
| AGXT | P21549 | G | R |
| AGXT | P21549 | D | N |
| AGXT | P21549 | S | F |
| AGXT | P21549 | S | P |
| AGXT | P21549 | R | C |
| AGXT | P21549 | R | H |
| AGXT | P21549 | I | T |
| AGXT | P21549 | I | M |
| ARSB | P15848 | S | F |
| ARSB | P15848 | T | M |
| ARSB | P15848 | R | Q |
| ARSB | P15848 | P | H |
| ARSB | P15848 | G | V |
| ARSB | P15848 | M | I |
| ARSB | P15848 | G | R |
| ARSB | P15848 | W | L |
| ARSB | P15848 | W | R |
| ARSB | P15848 | W | S |
| ARSB | P15848 | R | W |
| ARSB | P15848 | R | Q |
| ARSB | P15848 | c | R |
| ARSB | P15848 | Y | C |
| ARSB | P15848 | L | P |
| ARSB | P15848 | Q | R |
| ARSB | P15848 | G | R |
| ARSB | P15848 | W | C |
| ARSB | P15848 | R | Q |
| ARSB | P15848 | L | P |
| ARSB | P15848 | H | P |
| ARSB | P15848 | F | L |
| ARSB | P15848 | R | G |
| ARSB | P15848 | L | P |
| ARSB | P15848 | P | R |
| ASL | P04424 | R | C |
| ASL | P04424 | R | W |
| ASL | P04424 | V | M |
| ASL | P04424 | R | Q |
| ASL | P04424 | R | C |
| ASL | P04424 | R | C |
| BTK | Q06187 | L | P |
| BTK | Q06187 | K | R |
| BTK | Q06187 | S | F |
| BTK | Q06187 | K | E |
| BTK | Q06187 | K | E |
| BTK | Q06187 | F | S |
| BTK | Q06187 | K | R |
| BTK | Q06187 | L | S |
| BTK | Q06187 | L | W |
| BTK | Q06187 | T | I |
| BTK | Q06187 | T | P |
| BTK | Q06187 | Y | C |
| BTK | Q06187 | Y | S |
| BTK | Q06187 | Y | C |
| BTK | Q06187 | Y | N |
| BTK | Q06187 | I | N |
| BTK | Q06187 | V | D |
| BTK | Q06187 | V | F |
| BTK | Q06187 | P | S |
| BTK | Q06187 | F | V |
| BTK | Q06187 | L | R |
| BTK | Q06187 | Y | H |
| BTK | Q06187 | V | D |
| BTK | Q06187 | V | F |
| BTK | Q06187 | S | F |
| BTK | Q06187 | T | P |
| BTK | Q06187 | W | C |
| BTK | Q06187 | Q | H |
| BTK | Q06187 | C | G |
| BTK | Q06187 | C | S |
| BTK | Q06187 | C | G |
| BTK | Q06187 | C | R |
| BTK | Q06187 | C | Y |
| BTK | Q06187 | C | Y |
| BTK | Q06187 | L | P |
| BTK | Q06187 | G | R |
| BTK | Q06187 | Y | H |
| BTK | Q06187 | I | N |
| BTK | Q06187 | K | E |
| BTK | Q06187 | K | R |
| BTK | Q06187 | E | D |
| BTK | Q06187 | L | P |
| BTK | Q06187 | H | R |
| BTK | Q06187 | G | D |
| BTK | Q06187 | G | V |
| BTK | Q06187 | Y | D |
| BTK | Q06187 | M | R |
| BTK | Q06187 | L | P |
| BTK | Q06187 | L | V |
| BTK | Q06187 | M | T |
| BTK | Q06187 | C | F |
| BTK | Q06187 | C | W |
| BTK | Q06187 | D | V |
| BTK | Q06187 | C | F |
| BTK | Q06187 | C | R |
| BTK | Q06187 | C | Y |
| BTK | Q06187 | A | D |
| BTK | Q06187 | M | I |
| BTK | Q06187 | M | I |
| BTK | Q06187 | M | V |
| BTK | Q06187 | L | P |
| BTK | Q06187 | L | Q |
| BTK | Q06187 | L | R |
| BTK | Q06187 | R | G |
| BTK | Q06187 | R | Q |
| BTK | Q06187 | D | G |
| BTK | Q06187 | D | H |
| BTK | Q06187 | D | N |
| BTK | Q06187 | A | E |
| BTK | Q06187 | R | G |
| BTK | Q06187 | R | P |
| BTK | Q06187 | R | Q |
| BTK | Q06187 | N | K |
| BTK | Q06187 | C | F |
| BTK | Q06187 | C | S |
| BTK | Q06187 | V | F |
| BTK | Q06187 | V | E |
| BTK | Q06187 | S | P |
| BTK | Q06187 | G | D |
| BTK | Q06187 | L | P |
| BTK | Q06187 | R | G |
| BTK | Q06187 | R | K |
| BTK | Q06187 | R | S |
| BTK | Q06187 | F | S |
| BTK | Q06187 | R | P |
| BTK | Q06187 | R | W |
| BTK | Q06187 | W | L |
| BTK | Q06187 | P | L |
| BTK | Q06187 | P | S |
| BTK | Q06187 | E | D |
| BTK | Q06187 | E | K |
| BTK | Q06187 | L | P |
| BTK | Q06187 | S | R |
| BTK | Q06187 | S | Y |
| BTK | Q06187 | W | R |
| BTK | Q06187 | A | V |
| BTK | Q06187 | F | S |
| BTK | Q06187 | G | E |
| BTK | Q06187 | G | R |
| BTK | Q06187 | V | F |
| BTK | Q06187 | M | L |
| BTK | Q06187 | M | T |
| BTK | Q06187 | W | C |
| BTK | Q06187 | E | D |
| BTK | Q06187 | E | G |
| BTK | Q06187 | E | K |
| BTK | Q06187 | Y | S |
| BTK | Q06187 | S | P |
| BTK | Q06187 | S | Y |
| BTK | Q06187 | G | E |
| BTK | Q06187 | G | R |
| BTK | Q06187 | G | R |
| BTK | Q06187 | P | T |
| BTK | Q06187 | Y | C |
| BTK | Q06187 | Y | D |
| BTK | Q06187 | Y | S |
| BTK | Q06187 | T | P |
| BTK | Q06187 | A | D |
| BTK | Q06187 | Q | P |
| BTK | Q06187 | G | D |
| BTK | Q06187 | R | P |
| BTK | Q06187 | R | S |
| BTK | Q06187 | L | F |
| BTK | Q06187 | L | I |
| BTK | Q06187 | P | A |
| BTK | Q06187 | P | L |
| BTK | Q06187 | P | S |
| BTK | Q06187 | P | T |
| BTK | Q06187 | A | P |
| BTK | Q06187 | S | L |
| BTK | Q06187 | V | G |
| BTK | Q06187 | M | K |
| BTK | Q06187 | M | T |
| BTK | Q06187 | C | Y |
| BTK | Q06187 | W | S |
| BTK | Q06187 | R | C |
| BTK | Q06187 | R | H |
| BTK | Q06187 | T | I |
| BTK | Q06187 | F | L |
| BTK | Q06187 | F | S |
| BTK | Q06187 | L | P |
| BTK | Q06187 | L | R |
| BTK | Q06187 | L | P |
| BTK | Q06187 | L | P |
| CBS | P35520 | P | L |
| CBS | P35520 | R | W |
| CBS | P35520 | H | R |
| CBS | P35520 | P | R |
| CBS | P35520 | G | R |
| CBS | P35520 | P | S |
| CBS | P35520 | L | P |
| CBS | P35520 | K | N |
| CBS | P35520 | K | Q |
| CBS | P35520 | C | R |
| CBS | P35520 | A | V |
| CBS | P35520 | G | R |
| CBS | P35520 | R | C |
| CBS | P35520 | R | H |
| CBS | P35520 | R | L |
| CBS | P35520 | R | Q |
| CBS | P35520 | R | W |
| CBS | P35520 | M | V |
| CBS | P35520 | E | D |
| CBS | P35520 | E | D |
| CBS | P35520 | G | R |
| CBS | P35520 | I | M |
| CBS | P35520 | E | K |
| CBS | P35520 | P | L |
| CBS | P35520 | G | R |
| CBS | P35520 | G | R |
| CBS | P35520 | I | M |
| CBS | P35520 | A | T |
| CBS | P35520 | C | Y |
| CBS | P35520 | V | M |
| CBS | P35520 | E | K |
| CBS | P35520 | V | A |
| CBS | P35520 | T | M |
| CBS | P35520 | R | H |
| CBS | P35520 | A | T |
| CBS | P35520 | N | K |
| CBS | P35520 | D | N |
| CBS | P35520 | E | K |
| CBS | P35520 | T | M |
| CBS | P35520 | T | M |
| CBS | P35520 | T | R |
| CBS | P35520 | R | G |
| CBS | P35520 | R | K |
| CBS | P35520 | I | T |
| CBS | P35520 | P | L |
| CBS | P35520 | E | K |
| CBS | P35520 | G | R |
| CBS | P35520 | G | S |
| CBS | P35520 | V | A |
| CBS | P35520 | A | E |
| CBS | P35520 | A | V |
| CBS | P35520 | R | C |
| CBS | P35520 | R | H |
| CBS | P35520 | L | P |
| CBS | P35520 | G | S |
| CBS | P35520 | S | N |
| CBS | P35520 | S | N |
| CBS | P35520 | T | M |
| CBS | P35520 | V | M |
| CBS | P35520 | A | P |
| CBS | P35520 | R | C |
| CBS | P35520 | R | H |
| CBS | P35520 | C | Y |
| CBS | P35520 | V | M |
| CBS | P35520 | D | N |
| CBS | P35520 | R | Q |
| CBS | P35520 | K | E |
| CBS | P35520 | K | N |
| CBS | P35520 | M | I |
| CD40LG | P29965 | G | R |
| CD40LG | P29965 | A | E |
| CD40LG | P29965 | H | R |
| CD40LG | P29965 | V | A |
| CD40LG | P29965 | W | C |
| CD40LG | P29965 | W | G |
| CD40LG | P29965 | W | R |
| CD40LG | P29965 | G | E |
| CD40LG | P29965 | T | N |
| CD40LG | P29965 | L | P |
| CD40LG | P29965 | Y | C |
| CD40LG | P29965 | Y | H |
| CD40LG | P29965 | A | D |
| CD40LG | P29965 | Q | R |
| CD40LG | P29965 | T | I |
| CD40LG | P29965 | L | P |
| CD40LG | P29965 | T | N |
| CD40LG | P29965 | S | F |
| CD40LG | P29965 | G | V |
| CD40LG | P29965 | L | S |
| CD40LG | P29965 | A | P |
| CD40LG | P29965 | V | E |
| CD40LG | P29965 | G | A |
| CD40LG | P29965 | T | M |
| CD40LG | P29965 | G | D |
| CD40LG | P29965 | G | S |
| CD40LG | P29965 | L | S |
| CFTR | P13569 | A | E |
| CFTR | P13569 | V | F |
| CFTR | P13569 | G | V |
| CFTR | P13569 | E | Q |
| CFTR | P13569 | I | M |
| CFTR | P13569 | I | V |
| CFTR | P13569 | D | G |
| CFTR | P13569 | V | F |
| CFTR | P13569 | G | V |
| CFTR | P13569 | S | I |
| CFTR | P13569 | S | N |
| CFTR | P13569 | S | R |
| CFTR | P13569 | G | D |
| CFTR | P13569 | G | S |
| CFTR | P13569 | L | S |
| CFTR | P13569 | A | T |
| CFTR | P13569 | R | K |
| CFTR | P13569 | R | S |
| CFTR | P13569 | R | T |
| CFTR | P13569 | V | I |
| CFTR | P13569 | V | L |
| CFTR | P13569 | Y | N |
| CFTR | P13569 | Y | C |
| CFTR | P13569 | Y | D |
| CFTR | P13569 | Y | H |
| CFTR | P13569 | L | S |
| CFTR | P13569 | D | N |
| CFTR | P13569 | P | H |
| CFTR | P13569 | G | A |
| CFTR | P13569 | D | G |
| CFTR | P13569 | L | S |
| CFTR | P13569 | A | T |
| CFTR | P13569 | D | G |
| CFTR | P13569 | I | T |
| CFTR | P13569 | L | S |
| CFTR | P13569 | H | P |
| CFTR | P13569 | H | Q |
| CFTR | P13569 | G | D |
| CFTR | P13569 | G | R |
| CFTR | P13569 | L | P |
| CFTR | P13569 | D | N |
| CFTR | P13569 | T | S |
| CFTR | P13569 | R | C |
| CHEK2 | O96017 | R | C |
| CHEK2 | O96017 | R | H |
| CHEK2 | O96017 | R | C |
| CHEK2 | O96017 | R | H |
| CHEK2 | O96017 | R | G |
| CHEK2 | O96017 | R | Q |
| CHEK2 | O96017 | R | P |
| CHEK2 | O96017 | R | W |
| CHEK2 | O96017 | I | T |
| CHEK2 | O96017 | G | R |
| DCX | O43602 | S | R |
| DCX | O43602 | K | N |
| DCX | O43602 | R | H |
| DCX | O43602 | D | N |
| DCX | O43602 | G | E |
| DCX | O43602 | A | S |
| DCX | O43602 | R | H |
| DCX | O43602 | R | L |
| DCX | O43602 | D | H |
| DCX | O43602 | R | G |
| DCX | O43602 | L | R |
| DCX | O43602 | G | A |
| DCX | O43602 | R | S |
| DCX | O43602 | I | T |
| DCX | O43602 | Y | D |
| DCX | O43602 | Y | H |
| CYB5R3 | P00387 | S | P |
| CYB5R3 | P00387 | L | P |
| CYB5R3 | P00387 | A | V |
| CYB5R3 | P00387 | C | R |
| CYB5R3 | P00387 | C | Y |
| CYB5R3 | P00387 | R | Q |
| CYB5R3 | P00387 | L | P |
| CYB5R3 | P00387 | V | M |
| EDA | Q92838 | H | L |
| EDA | Q92838 | H | Y |
| EDA | Q92838 | G | C |
| EDA | Q92838 | G | D |
| EDA | Q92838 | G | V |
| EDA | Q92838 | W | G |
| EDA | Q92838 | G | R |
| EDA | Q92838 | G | W |
| EDA | Q92838 | D | H |
| EDA | Q92838 | G | S |
| EDA | Q92838 | F | S |
| EDA | Q92838 | C | Y |
| EDA | Q92838 | A | T |
| EDA | Q92838 | A | D |
| EDA | Q92838 | R | P |
| EDA | Q92838 | T | M |
| F8 | P00451 | G | V |
| F8 | P00451 | E | D |
| F8 | P00451 | I | T |
| F8 | P00451 | I | N |
| F8 | P00451 | A | P |
| F8 | P00451 | A | P |
| F8 | P00451 | P | L |
| F8 | P00451 | R | G |
| F8 | P00451 | R | L |
| F8 | P00451 | R | P |
| F8 | P00451 | R | Q |
| F8 | P00451 | V | M |
| F8 | P00451 | W | C |
| F8 | P00451 | W | S |
| F8 | P00451 | V | A |
| F8 | P00451 | V | E |
| F8 | P00451 | M | V |
| F8 | P00451 | T | A |
| F8 | P00451 | Q | R |
| F8 | P00451 | F | C |
| F8 | P00451 | F | I |
| F8 | P00451 | I | T |
| F8 | P00451 | W | L |
| F8 | P00451 | G | V |
| F8 | P00451 | D | A |
| F8 | P00451 | P | L |
| F8 | P00451 | P | S |
| F8 | P00451 | R | C |
| F8 | P00451 | R | G |
| F8 | P00451 | R | H |
| F8 | P00451 | R | G |
| F8 | P00451 | R | L |
| F8 | P00451 | R | P |
| F8 | P00451 | R | Q |
| F8 | P00451 | Q | P |
| F8 | P00451 | W | R |
| F8 | P00451 | R | T |
| F8 | P00451 | G | C |
| F8 | P00451 | G | S |
| G6PD | P11413 | V | L |
| G6PD | P11413 | H | R |
| G6PD | P11413 | A | G |
| G6PD | P11413 | I | T |
| G6PD | P11413 | V | M |
| G6PD | P11413 | Y | H |
| G6PD | P11413 | L | P |
| G6PD | P11413 | R | C |
| G6PD | P11413 | R | H |
| G6PD | P11413 | S | C |
| G6PD | P11413 | N | D |
| G6PD | P11413 | L | P |
| G6PD | P11413 | G | V |
| G6PD | P11413 | E | K |
| G6PD | P11413 | G | D |
| G6PD | P11413 | G | S |
| G6PD | P11413 | N | D |
| G6PD | P11413 | R | H |
| G6PD | P11413 | D | G |
| G6PD | P11413 | D | V |
| G6PD | P11413 | R | W |
| G6PD | P11413 | S | F |
| G6PD | P11413 | R | C |
| G6PD | P11413 | R | P |
| G6PD | P11413 | M | V |
| G6PD | P11413 | V | L |
| G6PD | P11413 | F | L |
| G6PD | P11413 | R | L |
| G6PD | P11413 | R | Q |
| G6PD | P11413 | R | G |
| G6PD | P11413 | E | K |
| G6PD | P11413 | S | F |
| G6PD | P11413 | T | S |
| G6PD | P11413 | D | H |
| G6PD | P11413 | R | H |
| G6PD | P11413 | V | M |
| G6PD | P11413 | E | K |
| G6PD | P11413 | Y | H |
| G6PD | P11413 | L | P |
| G6PD | P11413 | A | T |
| G6PD | P11413 | L | F |
| G6PD | P11413 | P | S |
| G6PD | P11413 | N | K |
| G6PD | P11413 | C | R |
| G6PD | P11413 | K | E |
| G6PD | P11413 | R | C |
| G6PD | P11413 | R | H |
| G6PD | P11413 | R | H |
| G6PD | P11413 | V | L |
| G6PD | P11413 | P | L |
| G6PD | P11413 | E | K |
| G6PD | P11413 | G | C |
| G6PD | P11413 | G | D |
| G6PD | P11413 | E | K |
| G6PD | P11413 | R | P |
| G6PD | P11413 | L | F |
| G6PD | P11413 | G | R |
| G6PD | P11413 | Q | H |
| G6PD | P11413 | R | C |
| G6PD | P11413 | R | H |
| G6PD | P11413 | R | H |
| G6PD | P11413 | G | V |
| GCH1 | P30793 | L | Q |
| GCH1 | P30793 | A | V |
| GCH1 | P30793 | L | P |
| GCH1 | P30793 | G | A |
| GCH1 | P30793 | R | P |
| GCH1 | P30793 | R | W |
| GCH1 | P30793 | G | V |
| GCH1 | P30793 | M | K |
| GCH1 | P30793 | M | R |
| GCH1 | P30793 | G | D |
| GCH1 | P30793 | D | N |
| GCH1 | P30793 | D | V |
| GCH1 | P30793 | I | K |
| GCH1 | P30793 | C | R |
| GCH1 | P30793 | C | W |
| GCH1 | P30793 | H | P |
| GCH1 | P30793 | H | P |
| GCH1 | P30793 | L | R |
| GCH1 | P30793 | S | T |
| GCH1 | P30793 | R | S |
| GCH1 | P30793 | Q | R |
| GCH1 | P30793 | R | H |
| GCH1 | P30793 | T | K |
| GCH1 | P30793 | V | I |
| GCH1 | P30793 | P | L |
| GCH1 | P30793 | G | E |
| GCH1 | P30793 | G | R |
| GCH1 | P30793 | M | I |
| GCH1 | P30793 | M | V |
| GCH1 | P30793 | M | V |
| GCH1 | P30793 | M | T |
| GCH1 | P30793 | K | R |
| GCH1 | P30793 | F | S |
| GCH1 | P30793 | R | W |
| GCH1 | P30793 | R | S |
| GLA | P06280 | L | P |
| GLA | P06280 | N | S |
| GLA | P06280 | G | R |
| GLA | P06280 | P | L |
| GLA | P06280 | P | S |
| GLA | P06280 | M | V |
| GLA | P06280 | H | R |
| GLA | P06280 | H | Y |
| GLA | P06280 | W | G |
| GLA | P06280 | R | L |
| GLA | P06280 | R | P |
| GLA | P06280 | R | S |
| GLA | P06280 | E | K |
| GLA | P06280 | E | Q |
| GLA | P06280 | M | V |
| GLA | P06280 | G | D |
| GLA | P06280 | Y | C |
| GLA | P06280 | L | P |
| GLA | P06280 | L | R |
| GLA | P06280 | I | T |
| GLA | P06280 | D | H |
| GLA | P06280 | D | Y |
| GLA | P06280 | D | G |
| GLA | P06280 | W | S |
| GLA | P06280 | A | V |
| GLA | P06280 | R | K |
| GLA | P06280 | R | T |
| GLA | P06280 | R | C |
| GLA | P06280 | R | H |
| GLA | P06280 | F | L |
| GLA | P06280 | F | S |
| GLA | P06280 | G | E |
| GLA | P06280 | L | P |
| GLA | P06280 | Y | S |
| GLA | P06280 | G | R |
| GLA | P06280 | A | P |
| GLA | P06280 | A | T |
| GLA | P06280 | G | V |
| GLA | P06280 | P | S |
| GLA | P06280 | S | N |
| GLA | P06280 | S | R |
| GLA | P06280 | A | T |
| GLA | P06280 | A | V |
| GLA | P06280 | W | C |
| GLA | P06280 | W | R |
| GLA | P06280 | G | V |
| GLA | P06280 | D | V |
| GLA | P06280 | L | V |
| GLA | P06280 | D | V |
| GLA | P06280 | G | D |
| GLA | P06280 | M | V |
| GLA | P06280 | P | T |
| GLA | P06280 | N | S |
| GLA | P06280 | Y | D |
| GLA | P06280 | N | D |
| GLA | P06280 | N | S |
| GLA | P06280 | W | R |
| GLA | P06280 | R | Q |
| GLA | P06280 | A | T |
| GLA | P06280 | D | N |
| GLA | P06280 | S | C |
| GLA | P06280 | W | C |
| GLA | P06280 | W | L |
| GLA | P06280 | I | N |
| GLA | P06280 | D | H |
| GLA | P06280 | D | N |
| GLA | P06280 | G | R |
| GLA | P06280 | P | L |
| GLA | P06280 | P | R |
| GLA | P06280 | G | A |
| GLA | P06280 | G | D |
| GLA | P06280 | N | S |
| GLA | P06280 | D | V |
| GLA | P06280 | P | R |
| GLA | P06280 | D | H |
| GLA | P06280 | D | N |
| GLA | P06280 | D | V |
| GLA | P06280 | M | I |
| GLA | P06280 | V | A |
| GLA | P06280 | N | K |
| GLA | P06280 | Q | E |
| GLA | P06280 | Q | H |
| GLA | P06280 | Q | H |
| GLA | P06280 | M | T |
| GLA | P06280 | W | C |
| GLA | P06280 | W | G |
| GLA | P06280 | A | D |
| GLA | P06280 | I | F |
| GLA | P06280 | M | I |
| GLA | P06280 | M | V |
| GLA | P06280 | S | F |
| GLA | P06280 | N | H |
| GLA | P06280 | N | K |
| GLA | P06280 | N | S |
| GLA | P06280 | R | Q |
| GLA | P06280 | D | Y |
| GLA | P06280 | V | E |
| GLA | P06280 | N | K |
| GLA | P06280 | N | Y |
| GLA | P06280 | Q | E |
| GLA | P06280 | Q | K |
| GLA | P06280 | G | A |
| GLA | P06280 | G | R |
| GLA | P06280 | W | R |
| GLA | P06280 | E | K |
| GLA | P06280 | R | Q |
| GLA | P06280 | R | W |
| GLA | P06280 | E | K |
| GLA | P06280 | G | R |
| GLA | P06280 | R | H |
| GLA | P06280 | G | D |
| GLA | P06280 | G | S |
| GLA | P06280 | A | D |
| GLA | P06280 | P | A |
| GLA | P06280 | P | T |
| GLA | P06280 | T | A |
| GUSB | P08236 | R | W |
| GUSB | P08236 | A | V |
| GUSB | P08236 | R | C |
| GUSB | P08236 | R | W |
| GUSB | P08236 | A | V |
| GUSB | P08236 | W | C |
| HBB | P68871 | E | K |
| HBB | P68871 | E | V |
| HBB | P68871 | E | G |
| HBB | P68871 | S | C |
| HBB | P68871 | A | V |
| HBB | P68871 | V | D |
| HBB | P68871 | V | I |
| HBB | P68871 | L | R |
| HBB | P68871 | W | R |
| HBB | P68871 | G | D |
| HBB | P68871 | V | M |
| HBB | P68871 | N | S |
| HBB | P68871 | D | H |
| HBB | P68871 | D | N |
| HBB | P68871 | E | A |
| HBB | P68871 | E | K |
| HBB | P68871 | A | D |
| HBB | P68871 | A | S |
| HBB | P68871 | G | D |
| HBB | P68871 | R | S |
| HBB | P68871 | L | P |
| HBB | P68871 | L | R |
| HBB | P68871 | L | V |
| HBB | P68871 | L | V |
| HBB | P68871 | V | D |
| HBB | P68871 | V | F |
| HBB | P68871 | V | L |
| HBB | P68871 | Y | F |
| HBB | P68871 | P | S |
| HBB | P68871 | P | T |
| HBB | P68871 | T | N |
| HBB | P68871 | F | L |
| HBB | P68871 | E | Q |
| HBB | P68871 | S | C |
| HBB | P68871 | F | S |
| HBB | P68871 | V | D |
| HBB | P68871 | K | E |
| HBB | P68871 | A | D |
| HBB | P68871 | V | M |
| HBB | P68871 | L | P |
| HBB | P68871 | G | V |
| HBB | P68871 | L | P |
| HBB | P68871 | A | D |
| HBB | P68871 | H | Y |
| HBB | P68871 | G | D |
| HBB | P68871 | A | D |
| HBB | P68871 | T | K |
| HBB | P68871 | L | P |
| HBB | P68871 | E | K |
| HBB | P68871 | L | P |
| HBB | P68871 | L | R |
| HBB | P68871 | H | N |
| HBB | P68871 | H | P |
| HBB | P68871 | H | Q |
| HBB | P68871 | L | P |
| HBB | P68871 | L | V |
| HBB | P68871 | H | Q |
| HBB | P68871 | V | G |
| HBB | P68871 | D | E |
| HBB | P68871 | N | Y |
| HBB | P68871 | F | L |
| HBB | P68871 | R | T |
| HBB | P68871 | V | M |
| HBB | P68871 | L | P |
| HBB | P68871 | C | F |
| HBB | P68871 | C | Y |
| HBB | P68871 | L | P |
| HBB | P68871 | A | D |
| HBB | P68871 | H | R |
| HBB | P68871 | H | Y |
| HBB | P68871 | E | K |
| HBB | P68871 | E | Q |
| HBB | P68871 | E | V |
| HBB | P68871 | T | I |
| HBB | P68871 | V | G |
| HBB | P68871 | Q | E |
| HBB | P68871 | A | D |
| HBB | P68871 | A | P |
| HBB | P68871 | A | V |
| HBB | P68871 | Q | K |
| HBB | P68871 | Q | P |
| HBB | P68871 | Q | R |
| HBB | P68871 | K | N |
| HBB | P68871 | A | P |
| HBB | P68871 | G | D |
| HBB | P68871 | A | P |
| HBB | P68871 | A | T |
| HBB | P68871 | A | V |
| HBB | P68871 | L | R |
| HBB | P68871 | H | D |
| HBB | P68871 | Y | C |
| HBB | P68871 | H | L |
| HBB | P68871 | H | P |
| HBB | P68871 | H | Q |
| HGD | Q93099 | L | P |
| HGD | Q93099 | E | A |
| HGD | Q93099 | W | G |
| HGD | Q93099 | Y | C |
| HGD | Q93099 | W | G |
| HGD | Q93099 | A | D |
| HGD | Q93099 | D | G |
| HGD | Q93099 | G | R |
| HGD | Q93099 | E | K |
| HGD | Q93099 | S | I |
| HGD | Q93099 | I | T |
| HGD | Q93099 | R | H |
| HGD | Q93099 | F | S |
| HGD | Q93099 | P | S |
| HGD | Q93099 | P | T |
| HGD | Q93099 | G | R |
| HGD | Q93099 | D | E |
| HGD | Q93099 | V | G |
| HGD | Q93099 | R | S |
| HGD | Q93099 | H | R |
| HPRT1 | P00492 | G | D |
| HPRT1 | P00492 | V | G |
| HPRT1 | P00492 | G | D |
| HPRT1 | P00492 | G | S |
| HPRT1 | P00492 | D | V |
| HPRT1 | P00492 | C | W |
| HPRT1 | P00492 | L | P |
| HPRT1 | P00492 | I | F |
| HPRT1 | P00492 | I | T |
| HPRT1 | P00492 | R | K |
| HPRT1 | P00492 | R | H |
| HPRT1 | P00492 | A | P |
| HPRT1 | P00492 | A | V |
| HPRT1 | P00492 | R | G |
| HPRT1 | P00492 | R | P |
| HPRT1 | P00492 | D | G |
| HPRT1 | P00492 | V | A |
| HPRT1 | P00492 | V | M |
| HPRT1 | P00492 | M | L |
| HPRT1 | P00492 | M | T |
| HPRT1 | P00492 | G | R |
| HPRT1 | P00492 | H | R |
| HPRT1 | P00492 | G | E |
| HPRT1 | P00492 | G | R |
| HPRT1 | P00492 | D | V |
| HPRT1 | P00492 | S | R |
| HPRT1 | P00492 | S | L |
| HPRT1 | P00492 | V | D |
| HPRT1 | P00492 | L | S |
| HPRT1 | P00492 | I | M |
| HPRT1 | P00492 | I | T |
| HPRT1 | P00492 | D | G |
| HPRT1 | P00492 | M | K |
| HPRT1 | P00492 | A | S |
| HPRT1 | P00492 | S | R |
| HPRT1 | P00492 | T | I |
| HPRT1 | P00492 | P | L |
| HPRT1 | P00492 | D | V |
| HPRT1 | P00492 | D | Y |
| HPRT1 | P00492 | I | T |
| HPRT1 | P00492 | V | A |
| HPRT1 | P00492 | D | E |
| HPRT1 | P00492 | D | N |
| HPRT1 | P00492 | Y | C |
| HPRT1 | P00492 | F | V |
| HPRT1 | P00492 | D | G |
| HPRT1 | P00492 | D | N |
| HPRT1 | P00492 | D | Y |
| HPRT1 | P00492 | H | D |
| HPRT1 | P00492 | H | R |
| HPRT1 | P00492 | C | Y |
| INSR | P06213 | G | V |
| INSR | P06213 | A | V |
| INSR | P06213 | A | D |
| INSR | P06213 | K | E |
| INSR | P06213 | R | W |
| INSR | P06213 | I | T |
| INSR | P06213 | R | Q |
| INSR | P06213 | R | W |
| INSR | P06213 | A | T |
| INSR | P06213 | A | E |
| INSR | P06213 | M | I |
| INSR | P06213 | R | Q |
| INSR | P06213 | R | Q |
| INSR | P06213 | R | W |
| INSR | P06213 | P | L |
| INSR | P06213 | E | D |
| INSR | P06213 | E | K |
| INSR | P06213 | W | L |
| INSR | P06213 | W | S |
| LMNA | P02545 | R | W |
| LMNA | P02545 | G | D |
| LMNA | P02545 | I | T |
| LMNA | P02545 | R | C |
| LMNA | P02545 | R | L |
| LMNA | P02545 | R | Q |
| LMNA | P02545 | R | W |
| LMNA | P02545 | K | N |
| LMNA | P02545 | R | C |
| LMNA | P02545 | R | H |
| LMNA | P02545 | R | P |
| LMNA | P02545 | T | K |
| LMNA | P02545 | L | P |
| NF2 | P35240 | N | Y |
| NF2 | P35240 | L | R |
| OAT | P04181 | N | K |
| OAT | P04181 | Y | H |
| OAT | P04181 | N | K |
| OAT | P04181 | Q | E |
| OAT | P04181 | C | F |
| OAT | P04181 | R | L |
| OAT | P04181 | R | T |
| OAT | P04181 | P | L |
| OAT | P04181 | Y | C |
| OAT | P04181 | R | P |
| OAT | P04181 | T | I |
| OAT | P04181 | A | P |
| OAT | P04181 | R | K |
| OAT | P04181 | H | Y |
| OAT | P04181 | V | M |
| OAT | P04181 | G | D |
| OAT | P04181 | G | A |
| OAT | P04181 | C | R |
| OAT | P04181 | L | P |
| OAT | P04181 | P | L |
| OAT | P04181 | L | F |
| OTC | P00480 | G | C |
| OTC | P00480 | R | C |
| OTC | P00480 | R | H |
| OTC | P00480 | L | F |
| OTC | P00480 | T | I |
| OTC | P00480 | L | P |
| OTC | P00480 | L | V |
| OTC | P00480 | N | I |
| OTC | P00480 | G | R |
| OTC | P00480 | Y | D |
| OTC | P00480 | M | T |
| OTC | P00480 | S | L |
| OTC | P00480 | L | P |
| OTC | P00480 | G | E |
| OTC | P00480 | G | D |
| OTC | P00480 | G | R |
| OTC | P00480 | E | K |
| OTC | P00480 | K | N |
| OTC | P00480 | S | R |
| OTC | P00480 | R | Q |
| OTC | P00480 | T | A |
| OTC | P00480 | R | T |
| OTC | P00480 | G | D |
| OTC | P00480 | A | E |
| OTC | P00480 | H | L |
| OTC | P00480 | H | R |
| OTC | P00480 | T | M |
| OTC | P00480 | D | G |
| OTC | P00480 | R | H |
| OTC | P00480 | L | S |
| OTC | P00480 | A | P |
| OTC | P00480 | R | P |
| OTC | P00480 | R | Q |
| OTC | P00480 | L | F |
| OTC | P00480 | I | T |
| OTC | P00480 | I | S |
| OTC | P00480 | N | S |
| OTC | P00480 | G | R |
| OTC | P00480 | H | Q |
| OTC | P00480 | H | R |
| OTC | P00480 | I | F |
| OTC | P00480 | I | M |
| OTC | P00480 | A | P |
| OTC | P00480 | Y | C |
| OTC | P00480 | T | M |
| OTC | P00480 | Q | H |
| OTC | P00480 | E | G |
| OTC | P00480 | H | L |
| OTC | P00480 | Y | C |
| OTC | P00480 | Y | D |
| OTC | P00480 | G | R |
| OTC | P00480 | G | V |
| OTC | P00480 | L | F |
| OTC | P00480 | S | R |
| OTC | P00480 | G | R |
| OTC | P00480 | D | V |
| OTC | P00480 | D | Y |
| OTC | P00480 | G | E |
| OTC | P00480 | G | R |
| OTC | P00480 | N | K |
| OTC | P00480 | L | P |
| OTC | P00480 | H | Y |
| OTC | P00480 | S | C |
| OTC | P00480 | M | I |
| OTC | P00480 | M | R |
| OTC | P00480 | S | R |
| OTC | P00480 | A | T |
| OTC | P00480 | A | V |
| OTC | P00480 | M | K |
| OTC | P00480 | H | Y |
| OTC | P00480 | Q | E |
| OTC | P00480 | P | A |
| OTC | P00480 | P | L |
| OTC | P00480 | P | R |
| OTC | P00480 | P | T |
| OTC | P00480 | T | I |
| OTC | P00480 | L | Q |
| OTC | P00480 | T | K |
| OTC | P00480 | H | P |
| OTC | P00480 | T | K |
| OTC | P00480 | D | G |
| OTC | P00480 | D | N |
| OTC | P00480 | T | A |
| OTC | P00480 | T | I |
| OTC | P00480 | W | L |
| OTC | P00480 | S | R |
| OTC | P00480 | M | T |
| OTC | P00480 | G | E |
| OTC | P00480 | R | Q |
| OTC | P00480 | R | W |
| OTC | P00480 | L | F |
| OTC | P00480 | H | L |
| OTC | P00480 | H | Q |
| OTC | P00480 | H | Y |
| OTC | P00480 | C | R |
| OTC | P00480 | C | Y |
| OTC | P00480 | L | F |
| OTC | P00480 | P | H |
| OTC | P00480 | R | L |
| OTC | P00480 | E | K |
| OTC | P00480 | R | G |
| OTC | P00480 | A | S |
| OTC | P00480 | V | L |
| OTC | P00480 | V | L |
| OTC | P00480 | S | P |
| OTC | P00480 | L | P |
| OTC | P00480 | T | K |
| OTC | P00480 | Y | C |
| OTC | P00480 | Y | D |
| OTC | P00480 | F | C |
| PAH | P00439 | T | I |
| PAH | P00439 | D | Y |
| PAH | P00439 | D | G |
| PAH | P00439 | D | V |
| PAH | P00439 | H | Y |
| PAH | P00439 | G | S |
| PAH | P00439 | D | H |
| PAH | P00439 | Y | N |
| PAH | P00439 | R | P |
| PAH | P00439 | R | Q |
| PAH | P00439 | R | W |
| PAH | P00439 | Q | P |
| PAH | P00439 | F | S |
| PAH | P00439 | I | T |
| PAH | P00439 | N | I |
| PAH | P00439 | N | S |
| PAH | P00439 | R | H |
| PAH | P00439 | H | D |
| PAH | P00439 | H | R |
| PAH | P00439 | G | A |
| PAH | P00439 | G | R |
| PAH | P00439 | P | T |
| PAH | P00439 | I | T |
| PAH | P00439 | I | V |
| PAH | P00439 | P | A |
| PAH | P00439 | R | L |
| PAH | P00439 | R | P |
| PAH | P00439 | V | L |
| PAH | P00439 | E | G |
| PAH | P00439 | E | Q |
| PAH | P00439 | V | A |
| PAH | P00439 | L | P |
| PAH | P00439 | H | R |
| PAH | P00439 | H | Y |
| PAH | P00439 | E | A |
| PAH | P00439 | Y | D |
| PAH | P00439 | N | D |
| PAH | P00439 | N | S |
| PAH | P00439 | P | T |
| PAH | P00439 | L | P |
| PAH | P00439 | L | P |
| PAH | P00439 | C | G |
| PAH | P00439 | G | V |
| PAH | P00439 | E | G |
| PAH | P00439 | D | V |
| PAH | P00439 | I | M |
| PAH | P00439 | P | R |
| PAH | P00439 | P | T |
| PAH | P00439 | V | I |
| PAH | P00439 | S | F |
| PAH | P00439 | S | P |
| PAH | P00439 | F | L |
| PAH | P00439 | T | P |
| PAH | P00439 | G | S |
| PAH | P00439 | F | S |
| PAH | P00439 | R | C |
| PAH | P00439 | R | H |
| PAH | P00439 | R | L |
| PAH | P00439 | L | F |
| PAH | P00439 | R | Q |
| PAH | P00439 | P | L |
| PAH | P00439 | V | A |
| PAH | P00439 | V | E |
| PAH | P00439 | V | L |
| PAH | P00439 | A | D |
| PAH | P00439 | G | V |
| PAH | P00439 | L | P |
| PAH | P00439 | L | F |
| PAH | P00439 | R | G |
| PAH | P00439 | R | Q |
| PAH | P00439 | R | W |
| PAH | P00439 | L | S |
| PAH | P00439 | L | V |
| PAH | P00439 | G | C |
| PAH | P00439 | A | T |
| PAH | P00439 | A | V |
| PAH | P00439 | R | P |
| PAH | P00439 | R | Q |
| PAH | P00439 | F | L |
| PAH | P00439 | H | L |
| PAH | P00439 | C | G |
| PAH | P00439 | I | L |
| PAH | P00439 | R | K |
| PAH | P00439 | R | S |
| PAH | P00439 | H | Y |
| PAH | P00439 | S | F |
| PAH | P00439 | K | E |
| PAH | P00439 | M | I |
| PAH | P00439 | M | V |
| PAH | P00439 | Y | C |
| PAH | P00439 | Y | D |
| PAH | P00439 | T | A |
| PAH | P00439 | T | N |
| PAH | P00439 | E | K |
| PAH | P00439 | P | L |
| PAH | P00439 | D | N |
| PAH | P00439 | I | F |
| PAH | P00439 | I | N |
| PAH | P00439 | R | C |
| PAH | P00439 | R | H |
| PAH | P00439 | F | C |
| PAH | P00439 | A | S |
| PAH | P00439 | A | V |
| PAH | P00439 | S | P |
| PAH | P00439 | Q | R |
| PAH | P00439 | I | V |
| PAH | P00439 | A | D |
| PAH | P00439 | A | V |
| PAH | P00439 | S | F |
| PAH | P00439 | L | P |
| PAH | P00439 | P | H |
| PAH | P00439 | I | T |
| PAH | P00439 | A | G |
| PAH | P00439 | A | T |
| PAH | P00439 | Y | C |
| PAH | P00439 | E | D |
| PAH | P00439 | F | L |
| PAH | P00439 | L | F |
| PAH | P00439 | C | S |
| PAH | P00439 | G | V |
| PAH | P00439 | D | Y |
| PAH | P00439 | K | R |
| PAH | P00439 | K | T |
| PAH | P00439 | A | T |
| PAH | P00439 | Y | C |
| PAH | P00439 | G | R |
| PAH | P00439 | G | V |
| PAH | P00439 | A | S |
| PAH | P00439 | A | T |
| PAH | P00439 | L | F |
| PAH | P00439 | L | V |
| PAH | P00439 | S | L |
| PAH | P00439 | S | P |
| PAH | P00439 | S | T |
| PAH | P00439 | C | G |
| PAH | P00439 | P | T |
| PAH | P00439 | P | H |
| PAH | P00439 | T | S |
| PAH | P00439 | Y | C |
| PAH | P00439 | T | M |
| PAH | P00439 | Y | C |
| PAH | P00439 | Y | H |
| PAH | P00439 | V | L |
| PAH | P00439 | V | M |
| PAH | P00439 | E | G |
| PAH | P00439 | D | A |
| PAH | P00439 | D | H |
| PAH | P00439 | A | G |
| PAH | P00439 | A | P |
| PAH | P00439 | A | V |
| PAH | P00439 | P | S |
| PAH | P00439 | R | Q |
| PAH | P00439 | R | W |
| PAH | P00439 | F | S |
| PAH | P00439 | R | P |
| PAH | P00439 | R | S |
| PAH | P00439 | Y | C |
| PAH | P00439 | D | N |
| PAH | P00439 | T | P |
| PAX6 | P26367 | N | S |
| PAX6 | P26367 | G | W |
| PAX6 | P26367 | R | G |
| PAX6 | P26367 | I | S |
| PAX6 | P26367 | I | V |
| PAX6 | P26367 | A | P |
| PAX6 | P26367 | I | S |
| PAX6 | P26367 | S | P |
| PAX6 | P26367 | R | Q |
| PAX6 | P26367 | V | L |
| PAX6 | P26367 | T | P |
| PAX6 | P26367 | G | V |
| PAX6 | P26367 | P | S |
| PAX6 | P26367 | A | E |
| PAX6 | P26367 | I | R |
| PAX6 | P26367 | P | R |
| PAX6 | P26367 | S | R |
| PAX6 | P26367 | V | D |
| PAX6 | P26367 | R | C |
| PDHA1 | P08559 | R | C |
| PDHA1 | P08559 | H | D |
| PDHA1 | P08559 | G | R |
| PDHA1 | P08559 | V | M |
| PDHA1 | P08559 | A | T |
| PDHA1 | P08559 | F | L |
| PDHA1 | P08559 | P | L |
| PDHA1 | P08559 | T | A |
| PDHA1 | P08559 | Y | N |
| PDHA1 | P08559 | D | A |
| PDHA1 | P08559 | R | G |
| PDHA1 | P08559 | R | Q |
| PDHA1 | P08559 | H | L |
| PDHA1 | P08559 | R | C |
| PDHA1 | P08559 | R | H |
| PDHA1 | P08559 | D | N |
| PKLR | P30613 | S | P |
| PKLR | P30613 | R | P |
| PKLR | P30613 | I | N |
| PKLR | P30613 | G | R |
| PKLR | P30613 | M | T |
| PKLR | P30613 | G | R |
| PKLR | P30613 | A | P |
| PKLR | P30613 | S | F |
| PKLR | P30613 | S | Y |
| PKLR | P30613 | V | D |
| PKLR | P30613 | I | T |
| PKLR | P30613 | L | P |
| PKLR | P30613 | G | V |
| PKLR | P30613 | R | C |
| PKLR | P30613 | E | Q |
| PKLR | P30613 | I | T |
| PKLR | P30613 | G | A |
| PKLR | P30613 | G | R |
| PKLR | P30613 | G | W |
| PKLR | P30613 | G | R |
| PKLR | P30613 | D | N |
| PKLR | P30613 | F | V |
| PKLR | P30613 | V | L |
| PKLR | P30613 | D | N |
| PKLR | P30613 | A | V |
| PKLR | P30613 | I | N |
| PKLR | P30613 | I | T |
| PKLR | P30613 | E | K |
| PKLR | P30613 | D | E |
| PKLR | P30613 | D | N |
| PKLR | P30613 | G | S |
| PKLR | P30613 | V | M |
| PKLR | P30613 | A | S |
| PKLR | P30613 | R | P |
| PKLR | P30613 | R | Q |
| PKLR | P30613 | D | H |
| PKLR | P30613 | G | A |
| PKLR | P30613 | G | D |
| PKLR | P30613 | I | F |
| PKLR | P30613 | K | N |
| PKLR | P30613 | A | D |
| PKLR | P30613 | I | T |
| PKLR | P30613 | R | C |
| PKLR | P30613 | R | H |
| PKLR | P30613 | N | D |
| PKLR | P30613 | G | D |
| PKLR | P30613 | V | F |
| PKLR | P30613 | S | I |
| PKLR | P30613 | T | M |
| PKLR | P30613 | E | G |
| PKLR | P30613 | D | N |
| PKLR | P30613 | A | T |
| PKLR | P30613 | N | K |
| PKLR | P30613 | N | S |
| PKLR | P30613 | A | D |
| PKLR | P30613 | A | V |
| PKLR | P30613 | T | A |
| PKLR | P30613 | T | I |
| PKLR | P30613 | Q | K |
| PKLR | P30613 | R | Q |
| PKLR | P30613 | R | W |
| PKLR | P30613 | E | A |
| PKLR | P30613 | E | D |
| PKLR | P30613 | A | T |
| PKLR | P30613 | G | D |
| PKLR | P30613 | A | V |
| PKLR | P30613 | V | M |
| PKLR | P30613 | A | G |
| PKLR | P30613 | A | V |
| PKLR | P30613 | T | A |
| PKLR | P30613 | R | H |
| PKLR | P30613 | S | F |
| PKLR | P30613 | R | W |
| PKLR | P30613 | R | Q |
| PKLR | P30613 | R | W |
| PKLR | P30613 | A | T |
| PKLR | P30613 | A | V |
| PKLR | P30613 | R | H |
| PKLR | P30613 | R | L |
| PKLR | P30613 | V | I |
| PKLR | P30613 | R | Q |
| PKLR | P30613 | G | R |
| PKLR | P30613 | R | C |
| PKLR | P30613 | R | Q |
| PKLR | P30613 | R | W |
| PKLR | P30613 | V | M |
| PKLR | P30613 | G | A |
| PKLR | P30613 | R | G |
| PKLR | P30613 | N | K |
| PRNP | P04156 | M | V |
| PRNP | P04156 | G | V |
| PRNP | P04156 | D | N |
| PRNP | P04156 | V | I |
| PRNP | P04156 | T | A |
| PRNP | P04156 | H | R |
| PRNP | P04156 | T | K |
| PRNP | P04156 | T | R |
| PRNP | P04156 | E | K |
| PRNP | P04156 | F | S |
| PRNP | P04156 | E | K |
| PRNP | P04156 | D | N |
| PRNP | P04156 | V | I |
| PRNP | P04156 | R | H |
| PRNP | P04156 | V | I |
| PRNP | P04156 | E | Q |
| PRNP | P04156 | Q | P |
| PRNP | P04156 | Q | R |
| PRNP | P04156 | E | K |
| RB1 | P06400 | K | Q |
| RB1 | P06400 | A | G |
| RB1 | P06400 | K | R |
| RB1 | P06400 | H | Y |
| RB1 | P06400 | S | L |
| RB1 | P06400 | V | E |
| RB1 | P06400 | L | P |
| RB1 | P06400 | R | W |
| RB1 | P06400 | L | P |
| RB1 | P06400 | Q | P |
| RB1 | P06400 | C | Y |
| RB1 | P06400 | C | R |
| SLC4A1 | P02730 | K | E |
| SLC4A1 | P02730 | E | K |
| SLC4A1 | P02730 | P | S |
| SLC4A1 | P02730 | A | D |
| SLC4A1 | P02730 | P | R |
| SOD1 | P00441 | A | S |
| SOD1 | P00441 | A | T |
| SOD1 | P00441 | A | V |
| SOD1 | P00441 | V | E |
| SOD1 | P00441 | L | Q |
| SOD1 | P00441 | G | R |
| SOD1 | P00441 | V | G |
| SOD1 | P00441 | V | M |
| SOD1 | P00441 | G | S |
| SOD1 | P00441 | E | G |
| SOD1 | P00441 | E | K |
| SOD1 | P00441 | G | R |
| SOD1 | P00441 | L | R |
| SOD1 | P00441 | L | V |
| SOD1 | P00441 | G | D |
| SOD1 | P00441 | G | S |
| SOD1 | P00441 | H | R |
| SOD1 | P00441 | F | C |
| SOD1 | P00441 | H | R |
| SOD1 | P00441 | H | Q |
| SOD1 | P00441 | E | K |
| SOD1 | P00441 | N | S |
| SOD1 | P00441 | L | R |
| SOD1 | P00441 | G | S |
| SOD1 | P00441 | D | Y |
| SOD1 | P00441 | L | F |
| SOD1 | P00441 | L | V |
| SOD1 | P00441 | G | R |
| SOD1 | P00441 | N | S |
| SOD1 | P00441 | A | V |
| SOD1 | P00441 | D | A |
| SOD1 | P00441 | D | V |
| SOD1 | P00441 | G | A |
| SOD1 | P00441 | G | C |
| SOD1 | P00441 | G | D |
| SOD1 | P00441 | G | R |
| SOD1 | P00441 | G | V |
| SOD1 | P00441 | E | G |
| SOD1 | P00441 | E | K |
| SOD1 | P00441 | D | G |
| SOD1 | P00441 | D | N |
| SOD1 | P00441 | I | F |
| SOD1 | P00441 | L | V |
| SOD1 | P00441 | G | V |
| SOD1 | P00441 | D | N |
| SOD1 | P00441 | I | M |
| SOD1 | P00441 | I | T |
| SOD1 | P00441 | I | T |
| SOD1 | P00441 | R | G |
| SOD1 | P00441 | D | V |
| SOD1 | P00441 | D | H |
| SOD1 | P00441 | L | S |
| SOD1 | P00441 | S | N |
| SOD1 | P00441 | N | K |
| SOD1 | P00441 | L | F |
| SOD1 | P00441 | L | S |
| SOD1 | P00441 | A | T |
| SOD1 | P00441 | V | G |
| SOD1 | P00441 | V | I |
| SOD1 | P00441 | I | T |
| SOD1 | P00441 | I | T |
| SRY | Q05066 | V | A |
| SRY | Q05066 | V | L |
| SRY | Q05066 | R | G |
| SRY | Q05066 | M | I |
| SRY | Q05066 | M | R |
| SRY | Q05066 | F | V |
| SRY | Q05066 | I | T |
| SRY | Q05066 | R | S |
| SRY | Q05066 | M | T |
| SRY | Q05066 | N | Y |
| SRY | Q05066 | I | M |
| SRY | Q05066 | S | G |
| SRY | Q05066 | G | E |
| SRY | Q05066 | G | R |
| SRY | Q05066 | L | H |
| SRY | Q05066 | K | I |
| SRY | Q05066 | P | R |
| SRY | Q05066 | F | S |
| SRY | Q05066 | A | T |
| SRY | Q05066 | Y | C |
| SRY | Q05066 | Y | F |
| SRY | Q05066 | P | R |
| SRY | Q05066 | R | W |
| TCF1 | P20823 | L | R |
| TCF1 | P20823 | K | E |
| TCF1 | P20823 | Y | C |
| TCF1 | P20823 | I | N |
| TCF1 | P20823 | P | T |
| TCF1 | P20823 | R | Q |
| TCF1 | P20823 | R | W |
| TCF1 | P20823 | V | M |
| TCF1 | P20823 | S | F |
| TCF1 | P20823 | H | Y |
| TCF1 | P20823 | K | N |
| TCF1 | P20823 | R | Q |
| TCF1 | P20823 | R | W |
| TCF1 | P20823 | A | T |
| TCF1 | P20823 | R | C |
| TCF1 | P20823 | R | H |
| TCF1 | P20823 | K | Q |
| TCF1 | P20823 | R | Q |
| TCF1 | P20823 | C | G |
| TCF1 | P20823 | L | M |
| TCF1 | P20823 | V | D |
| TCF1 | P20823 | T | M |
| TCF1 | P20823 | R | C |
| TCF1 | P20823 | R | W |
| TCF1 | P20823 | R | C |
| TCF1 | P20823 | R | H |
| THRB | P10828 | A | T |
| THRB | P10828 | R | W |
| THRB | P10828 | R | H |
| THRB | P10828 | R | C |
| THRB | P10828 | R | H |
| THRB | P10828 | G | R |
| THRB | P10828 | R | W |
| THRB | P10828 | Q | H |
| THRB | P10828 | K | I |
| THRB | P10828 | G | R |
| THRB | P10828 | G | S |
| THRB | P10828 | G | V |
| THRB | P10828 | G | E |
| THRB | P10828 | V | E |
| THRB | P10828 | T | I |
| THRB | P10828 | R | H |
| THRB | P10828 | M | V |
| THRB | P10828 | K | E |
| THRB | P10828 | C | R |
| THRB | P10828 | P | H |
| THRB | P10828 | P | S |
| THRB | P10828 | P | T |
| TNNI3 | P19429 | P | S |
| TNNI3 | P19429 | A | V |
| TNNI3 | P19429 | R | P |
| TNNI3 | P19429 | K | E |
| TNNI3 | P19429 | R | Q |
| TP53 | P04637 | M | T |
| TP53 | P04637 | A | P |
| TP53 | P04637 | C | Y |
| TP53 | P04637 | P | S |
| TP53 | P04637 | P | T |
| TP53 | P04637 | P | L |
| TP53 | P04637 | G | V |
| TP53 | P04637 | R | G |
| TP53 | P04637 | R | H |
| TP53 | P04637 | Y | C |
| TP53 | P04637 | R | G |
| TP53 | P04637 | R | H |
| TP53 | P04637 | R | L |
| TP53 | P04637 | H | R |
| TP53 | P04637 | Y | C |
| TP53 | P04637 | M | I |
| TP53 | P04637 | S | F |
| TP53 | P04637 | G | C |
| TP53 | P04637 | G | D |
| TP53 | P04637 | G | S |
| TP53 | P04637 | G | V |
| TP53 | P04637 | R | G |
| TP53 | P04637 | R | Q |
| TP53 | P04637 | R | W |
| TP53 | P04637 | L | P |
| TP53 | P04637 | E | K |
| TP53 | P04637 | V | L |
| TP53 | P04637 | R | C |
| TP53 | P04637 | R | G |
| TP53 | P04637 | R | H |
| TP53 | P04637 | C | Y |
| TP53 | P04637 | P | L |
| TP53 | P04637 | P | S |
| TP53 | P04637 | R | K |
| TP53 | P04637 | D | V |
| TP53 | P04637 | R | H |
| TP53 | P04637 | E | A |
| UROD | P06132 | G | E |
| UROD | P06132 | F | L |
| UROD | P06132 | P | L |
| UROD | P06132 | A | G |
| UROD | P06132 | A | S |
| UROD | P06132 | R | Q |
| UROD | P06132 | R | P |
| UROD | P06132 | G | D |
| UROD | P06132 | L | Q |
| UROD | P06132 | M | R |
| UROD | P06132 | E | K |
| UROD | P06132 | R | P |
| UROD | P06132 | L | F |
| UROD | P06132 | L | Q |
| UROD | P06132 | E | K |
| UROD | P06132 | S | F |
| UROD | P06132 | H | P |
| UROD | P06132 | F | L |
| UROD | P06132 | F | L |
| UROD | P06132 | P | S |
| UROD | P06132 | L | Q |
| UROD | P06132 | I | T |
| UROD | P06132 | G | E |
| UROD | P06132 | G | V |
| UROD | P06132 | L | R |
| UROD | P06132 | R | G |
| UROD | P06132 | G | S |
| UROD | P06132 | N | K |
| UROD | P06132 | Y | C |
| UROD | P06132 | G | R |
| UROD | P06132 | M | T |
| UROD | P06132 | R | H |
| UROD | P06132 | I | T |
| UROS | P10746 | V | F |
| UROS | P10746 | L | F |
| UROS | P10746 | Y | C |
| UROS | P10746 | P | L |
| UROS | P10746 | T | A |
| UROS | P10746 | A | V |
| UROS | P10746 | A | T |
| UROS | P10746 | C | R |
| UROS | P10746 | V | F |
| UROS | P10746 | V | A |
| UROS | P10746 | A | V |
| UROS | P10746 | I | T |
| UROS | P10746 | G | R |
| UROS | P10746 | G | W |
| UROS | P10746 | S | P |
| UROS | P10746 | I | S |
| UROS | P10746 | G | S |
| UROS | P10746 | T | M |
| VDR | P11473 | G | D |
| VDR | P11473 | H | Q |
| VDR | P11473 | K | E |
| VDR | P11473 | G | D |
| VDR | P11473 | F | I |
| VDR | P11473 | R | Q |
| VDR | P11473 | R | Q |
| VDR | P11473 | R | Q |
| VDR | P11473 | R | C |
| VHL | P40337 | S | L |
| VHL | P40337 | S | W |
| VHL | P40337 | S | W |
| VHL | P40337 | V | G |
| VHL | P40337 | F | I |
| VHL | P40337 | F | L |
| VHL | P40337 | F | S |
| VHL | P40337 | N | H |
| VHL | P40337 | N | S |
| VHL | P40337 | N | T |
| VHL | P40337 | R | P |
| VHL | P40337 | S | I |
| VHL | P40337 | S | N |
| VHL | P40337 | S | R |
| VHL | P40337 | P | S |
| VHL | P40337 | R | P |
| VHL | P40337 | V | L |
| VHL | P40337 | P | A |
| VHL | P40337 | P | L |
| VHL | P40337 | P | R |
| VHL | P40337 | P | S |
| VHL | P40337 | W | R |
| VHL | P40337 | W | S |
| VHL | P40337 | L | P |
| VHL | P40337 | G | D |
| VHL | P40337 | G | S |
| VHL | P40337 | Q | P |
| VHL | P40337 | Y | H |
| VHL | P40337 | L | R |
| VHL | P40337 | G | A |
| VHL | P40337 | T | P |
| VHL | P40337 | R | P |
| VHL | P40337 | S | C |
| VHL | P40337 | S | N |
| VHL | P40337 | S | R |
| VHL | P40337 | Y | H |
| VHL | P40337 | G | C |
| VHL | P40337 | G | R |
| VHL | P40337 | H | Q |
| VHL | P40337 | H | R |
| VHL | P40337 | H | Y |
| VHL | P40337 | L | V |
| VHL | P40337 | W | C |
| VHL | P40337 | L | P |
| VHL | P40337 | L | R |
| VHL | P40337 | F | L |
| VHL | P40337 | F | S |
| VHL | P40337 | D | G |
| VHL | P40337 | L | F |
| VHL | P40337 | V | L |
| VHL | P40337 | N | T |
| VHL | P40337 | F | C |
| VHL | P40337 | F | S |
| VHL | P40337 | A | T |
| VHL | P40337 | P | L |
| VHL | P40337 | Y | C |
| VHL | P40337 | Y | D |
| VHL | P40337 | T | I |
| VHL | P40337 | L | P |
| VHL | P40337 | L | V |
| VHL | P40337 | K | E |
| VHL | P40337 | R | G |
| VHL | P40337 | R | P |
| VHL | P40337 | R | Q |
| VHL | P40337 | C | F |
| VHL | P40337 | C | R |
| VHL | P40337 | C | W |
| VHL | P40337 | C | Y |
| VHL | P40337 | Q | H |
| VHL | P40337 | Q | R |
| VHL | P40337 | V | D |
| VHL | P40337 | V | F |
| VHL | P40337 | R | G |
| VHL | P40337 | R | Q |
| VHL | P40337 | R | W |
| VHL | P40337 | V | D |
| VHL | P40337 | V | F |
| VHL | P40337 | V | G |
| VHL | P40337 | Y | D |
| VHL | P40337 | L | P |
| VHL | P40337 | L | Q |
| VHL | P40337 | I | V |
| VHL | P40337 | L | P |
| VHL | P40337 | L | R |
| VHL | P40337 | E | K |
| VHL | P40337 | L | P |
| VHL | P40337 | L | Q |
| VHL | P40337 | L | V |
| VHL | P40337 | L | R |
| VHL | P40337 | R | W |

All the other mutations were obtained from HGMD. For the full list please contact the authors.
